# Morphometric analysis of postnatal lung development in the gray short‐tailed opossum (*Monodelphis domestica*): An ultrastructural study

**DOI:** 10.1002/ar.70122

**Published:** 2025-12-22

**Authors:** Kirsten Ferner

**Affiliations:** ^1^ Museum für Naturkunde Leibniz‐Institut für Evolutions‐ und Biodiversitätsforschung Berlin Germany

**Keywords:** lung development, marsupial, morphometry, TEM, ultrastructure

## Abstract

An ultrastructural morphometric analysis of the postnatal development of the lung in the gray short‐tailed opossum (*Monodelphis domestica*) has been conducted to evaluate the morphofunctional status of this poorly developed marsupial lung immediately following parturition. Allometric scaling of the volumes of the parenchymal components against body mass is carried out. In the parenchymal septa, volume proportions of pulmonary capillaries and solid septal tissue components (type I and type II epithelial cells, capillary endothelium, and interstitium) as well as alveolar and capillary surface densities are determined in transmission electron microscopic images using morphometry. Most parameters show a positive correlation with body mass over postnatal development. The lung of the neonate is at the late canalicular stage and consists of large terminal airspaces. The thick airspace septa are lined by cuboidal epithelium, interspersed with respiratory capillaries, and have a massive interstitial layer. A mature surfactant system is present at birth. The volume density of type II epithelial cells, which contain surfactant producing lamellar bodies, is highest in the late saccular stage, when septal growth is accelerated. The rapid development of the lung is indicated by an increase in the septal proportion of the parenchyma around postnatal day 4 and attainment of the saccular stage between day 4 and 7. The saccular stage, indicated by formation of a double capillary system, is characterized by repetitive steps of septation, increasing the number of saccular generations. By day 28 alveolarization starts and the process of microvascular maturation begins. The reduction of the interstitial tissue causes the two capillary beds to converge; finally, the two capillary layers begin to fuse, leading to a single capillary septum. In adults, capillaries and alveolar epithelia are the predominant septal components; the interstitium is greatly reduced. Despite the immaturity of the gray short‐tailed opossum lung, it appears to be structurally and quantitatively sufficient for gas exchange. The structural transformation of the septa is accompanied with an increase in gas exchange surface area and thinning of the diffusion barrier, in order to meet the metabolic requirements of the developing young.

## Graphical abstract

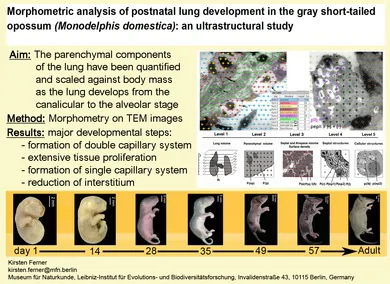

## 1 INTRODUCTION

In mammals the respiratory system needs to be adequately developed prior birth to take over the function of gas exchange, an event that is vital for continuity of life once the feto‐maternal connection has been severed (Burri et al., 2003). In the most immature marsupial neonates, where lung function is compromised for the first postnatal days, an alternative organ system such as the skin has to be temporarily recruited (e.g., Ferner, 2018, 2021a, 2025a; Frappell & MacFarlane, 2006; Frappell & Mortola, 2000; MacFarlane et al., 2021; MacFarlane & Frappell, 2001; Mortola et al., 1999).

The attainment of an efficiently functioning lung entails the development of a sufficient and structured gas exchange surface area, but also a quantitatively adequate surfactant system and a functional thin blood‐gas barrier (Gehr et al., 1978; Orgeig et al., 2004; Schittny, 2017). The formation of a functional blood‐gas barrier consists essentially in the conversion of the primitive cuboidal epithelium (pneumoblasts) into squamous type I cells while providing also a sufficient number of surfactant‐producing type II cells (Burri et al., 2003; Makanya et al., 2001). The thinning of the blood‐gas barrier results from an attenuation of type I cells and progressive approximation of the parenchymal capillaries to the epithelial layer due to the reduction of interstitial tissue (Burri, 1999; Burri et al., 2003; Mercurio & Rhodin, 1976). Furthermore, structural transformations, for example, formation of secondary septa, cause parenchymal air spaces to increase in number and finally lead to alveolarization of the lung (Burri, 1974). The microvascular maturation of the septa results in the conversion of the double capillary layer to a single capillary one, with an achievement of high diffusion capacities and high air space and capillary endothelial surface areas (Burri, 1974; Burri et al., 2003).

Mammalian lung development has mainly been studied in eutherian species, for example, in mice and rats, and can be categorized into five morphological stages (embryonic, pseudoglandular, canalicular, saccular and alveolar) based on characteristic morphology (Post & Copland, 2002; Schittny, 2017; Tschanz, 2007; Warburton et al., 2010). In the embryonic stage the lung bud is formed as an outpouching from the foregut and the development of the trachea, bronchi and bronchial tree begins. In the following pseudoglandular stage, the lungs resemble exocrine glands and extensive branching of the bronchial tree takes place including the formation of terminal bronchioles. In the canalicular stage respiratory bronchioles and alveolar ducts are formed, vascularization increases and capillaries approach the epithelium; type I and type II pneumocytes differentiate and surfactant production begins. Thus, prerequisites for a functional gas exchange have been developed. The following saccular stage is characterized by the formation of terminal saccules, which are separated by a double capillary septum, formation of the blood‐air barrier. Finally, in the alveolar stage, microvascular maturation leads to the formation of mature alveoli which are separated by single capillary septa.

The developmental steps that lead to a mature functional lung are complex and involve processes such as tissue proliferation with subdivision of the air spaces and increase in surface area, thinning of inter‐air space septa, and increase in capillary volume. The fact that different species of mammals are born with the lung at different levels of development means that the subsequent events leading to a mature lung structure also differ among species (Makanya et al., 2003). Despite this variation in event timing, the sequence of development steps resulting in final lung maturation remains highly conserved in mammals (Ferner et al., 2009, 2017; Mess & Ferner, 2010; Szdzuy et al., 2008; Tschanz, 2007).

Compared to eutherian neonates, which can be altricial or precocial, marsupials are generally in an earlier, nearly embryonic, stage of development at birth. In precocial mammals, such as the newborn elephant shrew or guinea pig, active life commences within minutes of birth and the lung is at an advanced alveolar stage of development (Szdzuy et al., 2008). The lung of newborn altricial eutherians is at the late saccular stage (Ferner et al., 2017; Mess & Ferner, 2010; Szdzuy et al., 2008). Marsupial neonates are similar in development to a late eutherian fetus (Fujii et al., 2020; Wang et al., 2009).

Previous investigations have established that the lung of newborn marsupials is either at the canalicular stage (e.g., Burri et al., 2003; Ferner, 2018, 2021a; Makanya et al., 2001, 2003, 2007; Modepalli et al., 2018; Simpson et al., 2011) or saccular stage (e.g., Gemmell & Nelson, 1988; Krause & Leeson, 1975; Mess & Ferner, 2010; Runciman et al., 1996, 1998a). The terminal air spaces in the lung of newborn marsupials are lined with a respiratory epithelium of type I and type II cells. The latter produce surfactant (Gemmell & Little, 1982; Orgeig et al., 2004; Ribbons et al., 1989). The terminal air spaces are well vascularized with capillaries which lie subjacent to the respiratory epithelium on each side of the thick septum (Makanya et al., 2003). Development, from the canalicular or early saccular stage to an alveolar lung, takes place over an extended period in an air breathing environment (35–180 days) (Buaboocha & Gemmell, 1997; Gemmell, 1986; Krause & Leeson, 1975; Runciman et al., 1998a; Simpson et al., 2011).

Morphometric and allometric analysis have repeatedly been used as a tool to evaluate the diverse quantitative morphological changes in the postnatally developing mammalian lung, but most of these studies were carried out on eutherians, including the rat (Burri et al., 1974), the sheep (Davies et al., 1988), and humans (Langston et al., 1984; Zeltner et al., 1987). There are only a few studies on marsupial lung morphometry so far. The most detailed morphometric studies on the developing marsupial lung have been carried out on the tammar wallaby (*Notamacropus eugenii*) (Runciman et al., 1998a, 1998b, 1999) and the quokka (*Setonix brachyurus*) (Burri et al., 2003; Makanya et al., 2003).

Runciman et al. (1999) have described three stages of postnatal lung development in the tammar wallaby; first, a period of initial septal tissue proliferation; second, an increase in air space surface area; and a final maturation of the parenchymal septa. In the quokka, a brief canalicular stage, a very prolonged saccular stage with repetitive steps of septation, resulting in a drastic increase in lung parenchyma, and finally the attainment of the alveolar stage, resulting in the conversion of the double capillary layer into a single one, has been described (Burri et al., 2003).

The prolonged lung development of the newborn gray short‐tailed opossum offers a great opportunity to understand lung development. In contrast to eutherian mammals, where the canalicular stage of lung development is confined to the prenatal period, marsupials encounter this canalicular stage postnatally and the entire process of postnatal lung development occurs in a ventilated functioning state. In the current study, the parenchymal components of the lung are quantified and scaled against body mass as the lung develops from the canalicular to the mature adult organ. This study augments a sister report in which the coarse parameters of the lung are analyzed by morphometry and allometry using optical serial sections of μCT scans (Ferner, 2025b).

## 2 MATERIALS AND METHODS

### 2.1 Experimental animals

The gray short‐tailed opossums (*Monodelphis domestica*) used for this study were obtained from a laboratory colony established at the Museum für Naturkunde Berlin. A total of 33 animals were included in this study. After controlled mating, the females were checked for offspring when approaching full‐term (13–14 days). Gray short‐tailed opossum young at ages of 0 days (<24 h), 4, 7, 11, 14, 21, 28, 35, 49, and 57 days postpartum (days pp) and adults were collected for this study. At the time of collection, the animals were weighed to 0.01 g and euthanized by anesthetic overdose with isoflurane under animal ethics permit approved by the Animal Experimentation Ethics Committee (registration number: T0202/18).

Details of the specimens used in this study are summarized in the supplement (Table S1, Supporting Information).

### 2.2 Lung fixation

Young ranging from neonate to 28 days postpartum (days pp) were decapitated to allow fixative inflow into the airways and lungs, and the whole animals were immersed in Karnovsky fixative (2 g paraformaldehyde, 25 mL distilled water, 10 mL 25% glutaraldehyde, 15 mL 0.2M phosphate buffer) for 2 h. In small age stages from neonate to 11 days pp the upper part of the trunk was cut in two halves and in older stages (14–28 days pp) the lung was removed under a dissecting microscope. This was achieved by ascertaining that the lobes were closely applied to the walls of the pleural cavity. For the larger age stages (35 days pp–adult), fixation of the lung was accomplished by intratracheal infusion. A pneumothorax was induced by making a ventromedian incision in the abdominal wall and puncturing the diaphragm. The lungs were removed from the thoracic cavity by careful dissection and volumes of the right lung together with the accessory lobe and that of the left lung estimated by fluid displacement (Scherle, 1970). The volume measurement of the lungs from 0 to 28 days pp were obtained by Cavalieri principle in μCT‐scans.

The lung samples were further fixed in 2.5% glutaraldehyde buffered in 0.2M cacodylate (pH 7.4) for 24 h and then rinsed with 0.1M cacodylate buffer, postfixed in 1% osmium tetroxide, dehydrated through ascending concentrations of ethanol and embedded in epoxy resin (Araldite).

In small ontogenetic stages (0–11 days pp) the two parts of the upper body were embedded in total. From 14 to 28 days pp the entire lung was cut in five pieces and from 35 days pp to adult five samples from the right lung were taken. One sample was taken from the cranial, middle and accessory lobes respectively and two samples were taken from the caudal lobe. The sampling procedure ensures that all parts of the lung were included in the ultrastructural analysis.

### 2.3 μCT imaging

For visualization of the lungs before sectioning and estimation of the lung volumes in small ontogenetic stages, the specimens were scanned by micro‐CT. Processing and scanning details for the specimens investigated in this study were previously published (Ferner, 2024a, 2024b, 2025b; Ferner & Mahlow, 2023). The specimens processed for electron microscopy and embedded in Araldite could be scanned with a Phoenix nanotom X‐ray machine (Waygate Technologies, Baker Hughes, Wunstorf, Germany; equipment reference ID SCR_022582) without staining. The post fixation with osmium tetroxide during the TEM processing produced scans with strong contrast. The Araldite blocks were fixed on a glass plate with glue to avoid moving during the scans. Three blocks at a time could be mounted and scanned consecutively with the best resolution for each sample, respectively. The scanning results of the Araldite blocks depended on proper fixation of the TEM specimens. The μCT scans of the Araldite blocks offer an opportunity to check the quality of TEM specimens before performing expensive and time‐consuming ultrathin sections. More importantly, it offers the possibility to estimate the reference lung volume of very small animals, in which conventional methods of lung volume measurement, for example, fluid displacement, prove to be difficult or impossible without destroying the lung.

### 2.4 Tissue processing for transmission electron microscopy

The specimen embedded in Araldite were sectioned in semithin (500 nm) and ultrathin (90 nm) sections using a Leica Ultramicrotom (Leica EMUC7). In small ontogenetic species (0–11 days pp), where the whole torso or lungs were embedded, the entire lungs were sectioned following the fractionator principle in eight sections. In larger stages (14 days pp–adult) sections were obtained from five blocks per animal. The sections were picked up on 200‐mesh copper grids, counterstained with lead citrate and uranyl acetate replacement stain (Electron Microscopy Sciences) and viewed on a LEO TEM 906 E connected to a Wide‐angle Dual Speed CCD Camera (2K‐CCD‐Camera for EM 10‐902).

### 2.5 Lung morphometry

The micro‐CT data were analyzed as two‐dimensional (2D) serial images using the software Volume Graphics Studio Max Version 3.5 (Volume Graphics GmbH, Heidelberg, Germany). Lung volume V(L) was determined for reference to translate relative stereological parameters (e.g., volume, surface density) into absolute values using Cavalieri principle and point counting methods (Hsia et al., 2010; Tschanz et al., 2014).

Since the whole lung was scanned, the length of the total lung was known. After a random start an optical fractionator, resulting from total length divided by 8, resulted in the section thickness. For each lung eight images representing the whole lung were taken at the same magnification. A morphometric analyzing program STEPanizer (Tschanz et al., 2011) was used to determine lung volume. Total lung volume V(L) was calculated using the formula proposed by Hsia et al. (2010),

$$V\left(L\right)=\Sigma P\;*\;d^{2}*\;t,$$

where *P* is the number of points falling on lung tissue, *d* is the distance between adjacent grid points, and *t* is the distance between the selected lung sections.

For the volume densities (reported as Vv) the parameter examined is always indicated before the comma and the reference space is indicated following the comma.

To examine the structural changes in the developing lung, the volume densities of the lung parenchyma Vv(p,L) and of its components, the air spaces Vv(a,p), the septa Vv(s,p), and that of the non‐parenchyma Vv(np,L) and its components [blood vessels Vv(v,np), bronchioles Vv(b,np), and connective tissue Vv(t,np)] have been previously estimated by point counting methods using light microscopy (Ferner, 2025b).

For ultrastructural analysis ultrathin sections from eight samples (0–11 days pp) or five blocks per animal were used for TEM morphometry. Starting at a random position on the top left‐hand corner of the grid, photographs of the quadrants with parenchymatous tissue (septa) were taken in a consecutive manner, while scoring all the quadrants covered by the air spaces. Five (0–11 days pp) or eight (14 days pp–adult) photographs per section were obtained at magnifications of 2000×, making a total of 40 per animal. A morphometric analyzing program STEPanizer (Tschanz et al., 2011) was used to determine the volume densities of septal lung components, such as the capillary lumen Vv(c,s), erythrocytes Vv(e,s), type I cells Vv(ep1,s), type II cells Vv(ep2,s), interstitium Vv(I,s), the endothelium Vv(en,s) as well as alveolar Sv(a,s) and capillary Sv(c,s) surface areas. To score point and intersection counts, a test grid with lines and points was used and the data fed directly into the STEPanizer program for calculation of totals. Microsoft Excel 2019 (Microsoft Excel, Microsoft Corporation, USA) was then used to compute the various parameters.

Multiplying the volume densities of the septal components with the volume densities of parenchyma and septum and the lung volume produced the absolute volumes of capillaries V(c), erythrocytes V(e), type I cells V(ep1), type II cells V(ep2), interstitium V(i), and the endothelium V(en) (Figure 1). Sv(a) and Sv(c) multiplied by the volume densities of lung parenchyma and alveolar space and septa respectively and by the absolute lung volume yielded the absolute surface areas S(a) and S(c). Other parameters estimated included capillary loading (Vc/Sa), the estimate of septal thickness (2Vs/Sa), and the surface to volume ratio of the air spaces (S(a)/V(a)).

**FIGURE 1:**
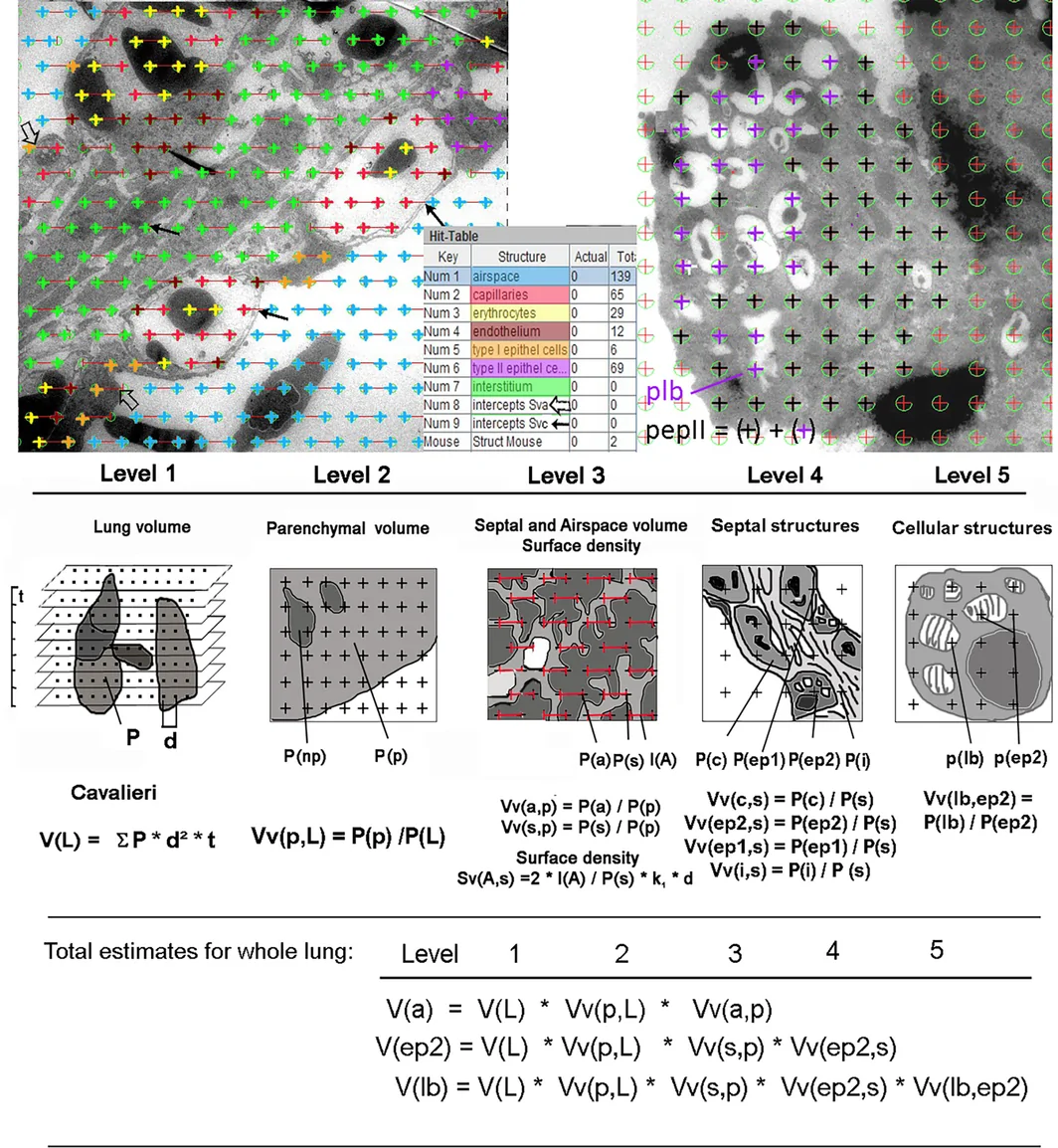
Estimating morphometric parameters of lung parenchyma and its components using multistage stratified sampling at five levels of increasing magnification. The parameter estimated at one level becomes the reference parameter at the next higher level. This approach allows calculation of total estimates pertaining to the whole lung and permits efficient sampling. Level 1 (magnification 50×) sections are overlaid with a simple point grid, allowing estimation of total lung volume (V(L): estimating total lung volume by measuring the area of lung surfaces (Ai) for all tissue slices i, and multiplying the summed areas by the slice thickness (t)). At level 2 (magnification 100×) volume fractions of parenchyma and non‐parenchyma (Vv(p,L), Vv(np,L)) of the lung can be estimated using a point grid (counting, on Cavalieri slice surfaces, points hitting coarse parenchyma (airspace, septum) and non‐parenchymal (bronchi and vessels >30 μm, connective tissue). At level 3 (magnification 200×), sections are overlaid with a multipurpose test system comprising a set of test line segments within an unbiased counting frame. Septal and airspace volume densities (Vv(s,p), Vv(a,p)) and surface density (Sv(a)) can be estimated. At level 4 (magnification 2000×) volume densities of the septal components (Vv(c,s), Vv(ep2,s), Vv(ep1,s), Vv(i,s)) are estimated. The two screen shots (upper panel—left: neonatal septum; right: type II epithelial cell) show how point and intercept counting was performed using the morphometric analyzing program Stepanizer. Differential counts of points (P) hitting on septal components (e.g., capillaries, red; interstitium, green; type I epithelial cells, orange; type II epithelial cells, purple) in relation to all points hitting the septum P(s) yield their respective volume densities per unit volume of septum (middle panel) and finally the total volumes (lower panel). The intercepts are counted when the intercept lines (red) cross the air space septa (Sv(a)) and blood‐air barrier of capillaries (Sv(c)). At level 5 (magnification 10,000×) the volume density of lammelar bodies within alveolar epithelial II cells (Vv(lb,ep2) have been estimated. A schematic representation and equations for volume estimation show the work flow of morphometric analysis (modified from Hsia et al., 2010). a, airspace; c, capillary; ep1, alveolar epithelial cell 1; ep2, alveolar epithelial cell 2; i, interstitium; L, lung; lb, lamellar body; p, parenchyma; s, septum. Part of the figure has been published previously in Ferner (2025b).

To assess differences in surfactant production during the course of lung development, the volume density of lamellar bodies Vv(lb,ep2) within type II cells was measured. For this purpose, 40 photographs of type II cells per animal were obtained at a magnification of 10,000×, and point counts were carried out with the STEPanizer program. By multiplying with the volume densities of parenchyma, septum, and type II cells and total lung volume, the absolute volume of lamellar bodies V(lb) in the lung was estimated.

Measurement of the thickness of the blood‐gas barrier (i.e., epithelium, interstitium, endothelium, and plasma) was performed using a scaled ruler with the Image J program (Image J software, National Institutes of Health, USA). The diffusion barrier was measured in 20 photographs per animal, obtained at a magnification of 46,000×. These measurements correspond to the harmonic mean barrier thickness *τ*_hb_.

The arithmetic mean barrier thickness barrier *τ*_t_, was estimated from the formula,

$$\tau_{t}=V\left(\mathrm{st}\right)/\left(S\left(a\right)+S\left(c\right)\right)/2,$$

where V(st) is the volume of the solid components of the septal tissue (i.e., V(en) + V(i) + V(ep)) and S(a) and S(c) are the air space and capillary surface areas, respectively.

### 2.6 Statistical analysis

All data of age groups are presented as means ± SEM. Differences in the weight specific lung volumes, volume proportions of the various components of the lung, surface densities, and body mass‐normalized surface areas, arithmetic mean barrier thickness (tt), the measured mean barrier thickness, estimate of septal thickness (2V(s)/S(a)), capillary loading (V(c)/S(a)), and surface to volume ratio (S(a)/V(a)) were normally distributed and statistically tested by one‐way analysis of variance (ANOVA). When the ANOVA showed that there is significant variance between age groups, the Tukey test was used to test each age groups against each other to identify significant differences between pairs. The ANOVA and Tukey test (Bärlocher, 1999) were calculated with Microsoft Excel 2019. All values were considered significant at the *p* < 0.05 level. SigmaPlot version 9.0 (Systat Software, Inc.) was used to perform least‐squares regression analysis whereby the coefficient of variation *r* was estimated to determine the goodness‐of‐fit. All data were log‐transformed and the power equation,

$$\log_{10}Y=\log a+b\log_{10}X,$$

where *Y* is the dependent parameter (e.g., volume or surface area), *X* is the predictor variable (body mass), *a* is the normalization constant (intercept on a log–log scale), and *b* is the scaling exponent (slope on a log–log scale), was used. To calculate the scaling exponent and then the relevant allometric equations were obtained using the allometric equation,

$$Y={\mathrm{aX}}^{b}.$$

Regression coefficients and allometric equations are provided in the figures.

## 3 RESULTS

### 3.1 The parenchymal morphology

The changing morphology of the septal tissue components of the lung parenchyma is shown in Figures 2 and 3. In the late canalicular stage, from neonate to 4 days pp (Figure 2a,b), a thick interairspace septum with abundant cellular and acellular connective tissue components exists. Typical for the canalicular stage is that the large terminal airspaces are lined by a cuboidal epithelium alternating with several respiratory capillaries which provide a mature blood‐gas barrier. The saccular stage is encountered by 7 days pp, characterized by a continuous double capillary septum. The saccular stage persists until 21 days pp. Newly formed double capillary systems have less abundant interstitium and become increasingly thinner (Figure 2c–f) during that period. By 28 days pp the process of microvascular maturation begins and alveolarization starts (Figure 3a). Details of microvascular maturation, that is, the transformation from double to single capillary septum are presented in Figure 4a–c. The reduction of the interstitial tissue causes the two capillary beds to converge. Finally, the two capillary layers begin to fuse (Figure 4a). During a transition period between 28 and 35 days pp, persisting double capillary and newly formed single capillary septa can be found at the same time (Figures 3a,b and 4b). Further reduction of the interstitial tissue and remodeling of the remaining tissue components result in septa in which a single capillary layer is predominant (Figure 4c). The process of microvascular maturation is finished during the alveolar period (Figure 3c,d) so that in adults capillaries and alveolar epithelia are the predominant septal components and the interstitium is greatly reduced (Figure 3e). Compared to earlier developmental stages, in adults the capillary lumina of the septa increase to allow for a higher load of erythrocytes.

**FIGURE 2:**
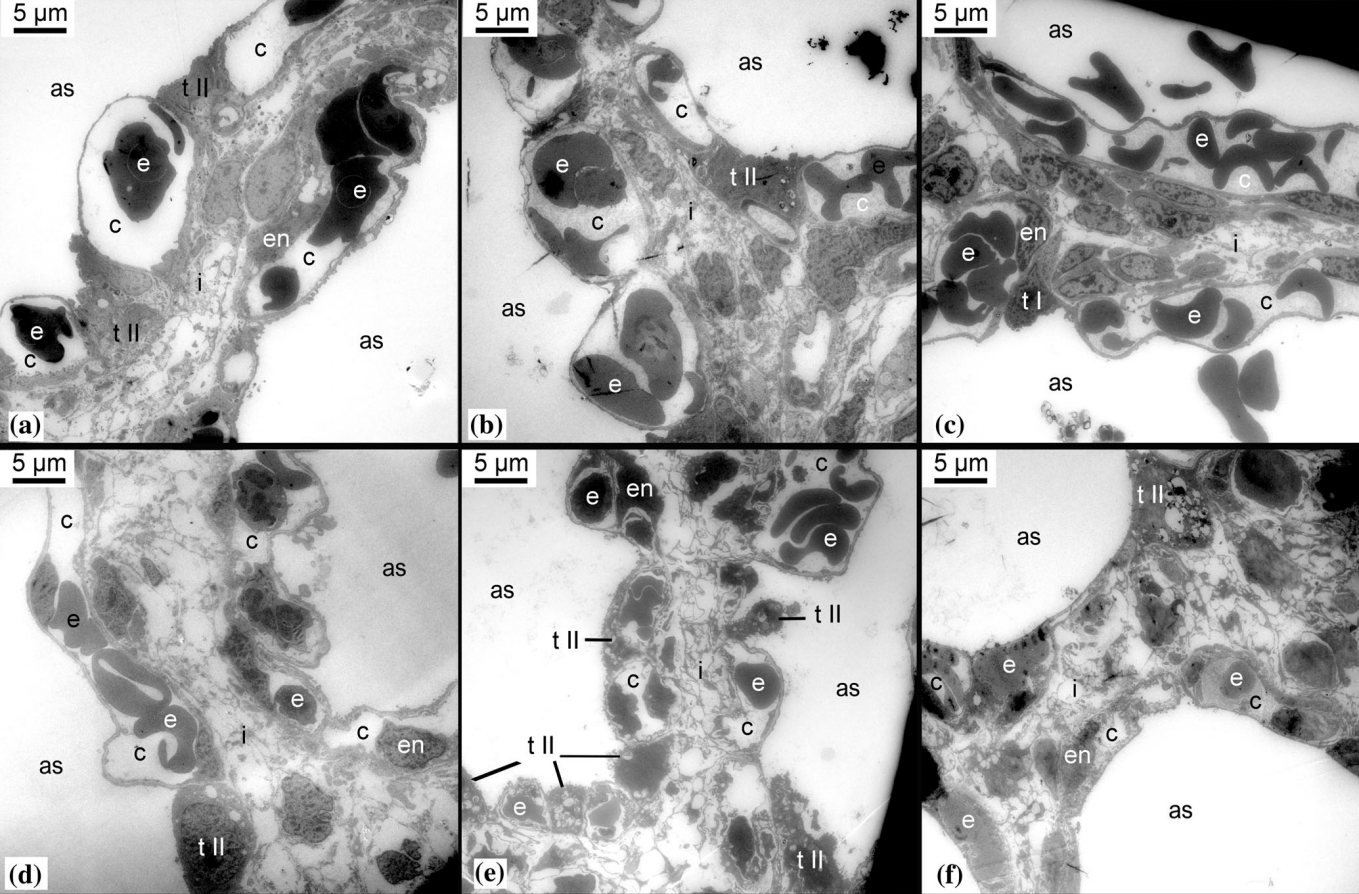
Transmission electron microscopic images of the lung parenchymal septum of the *Monodelphis domestica* lung in the neonate (a), at 4 (b), 7 (c), 11 (d), 14 (e) and 21 (f) days pp. In the newborn lung, the septa have a thick interstitium consisting mainly of connective tissue and fibroblasts. The septum is characterized by stretches of respiratory capillaries alternating with epithelial cells. No continuous double capillary septum is present yet, indicating that the lung is at the canalicular stage of lung development. Between 4 and 7 days pp the lung enters the saccular stage. By 7 days pp a continuous double capillary septum can be seen. During the mid and late saccular stage, from 11 to 21 days pp, the septa become thinner due to a reduction of the interstitial layer. Magnification: 2000×. days pp, days postpartum; as, airspace; c, capillary; e, erythrocyte; en, endothelial cell; i, interstitium; t I, epithelial type I cell; t II, epithelial type II cell.

**FIGURE 3:**
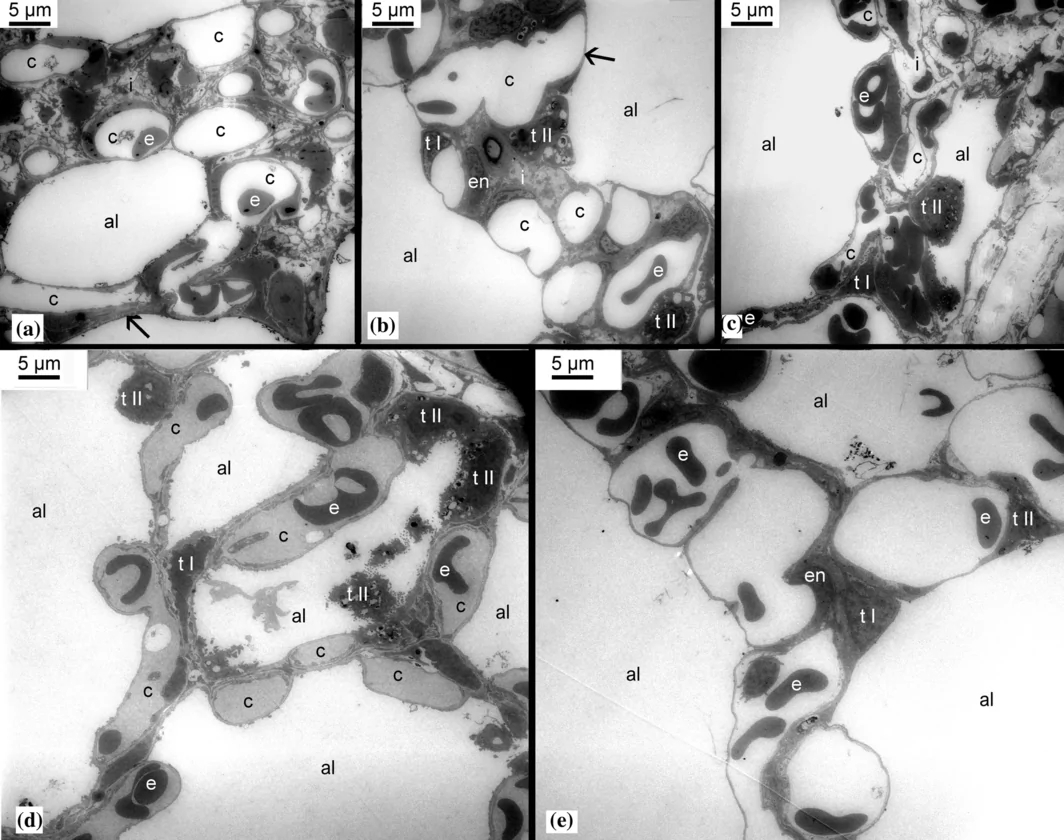
Transmission electron microscopic images of the lung parenchymal septum of the *Monodelphis domestica* lung at 28 (a), 35 (b), 49 (c), 57 (d) days pp and in the adult (e). Starting by 28 days pp first alveoli (al) are present, indicating the transition from the saccular to the alveolar stage. A few first single capillary septa can be seen (indicated by arrow), but the majority of septa are still double capillary. By 35 days pp microvascular maturation results in the transformation from double to single capillary septa (indicated by arrow). During the period of continued alveolarization single capillary septa are predominant. The interstitium is greatly reduced and the septa become thinner. In the adult only thin stretches of interstitium can be found, a single capillary spanning the entire septum is prevailing. The septa increase in thickness again due to expansion of the capillary lumina. Magnification: 2000×. days pp, days postpartum; al, alveolar space; c, capillary; e, erythrocyte; en, endothelial cell; i, interstitium; t I, epithelial type I cell; t II, epithelial type II cell.

**FIGURE 4:**
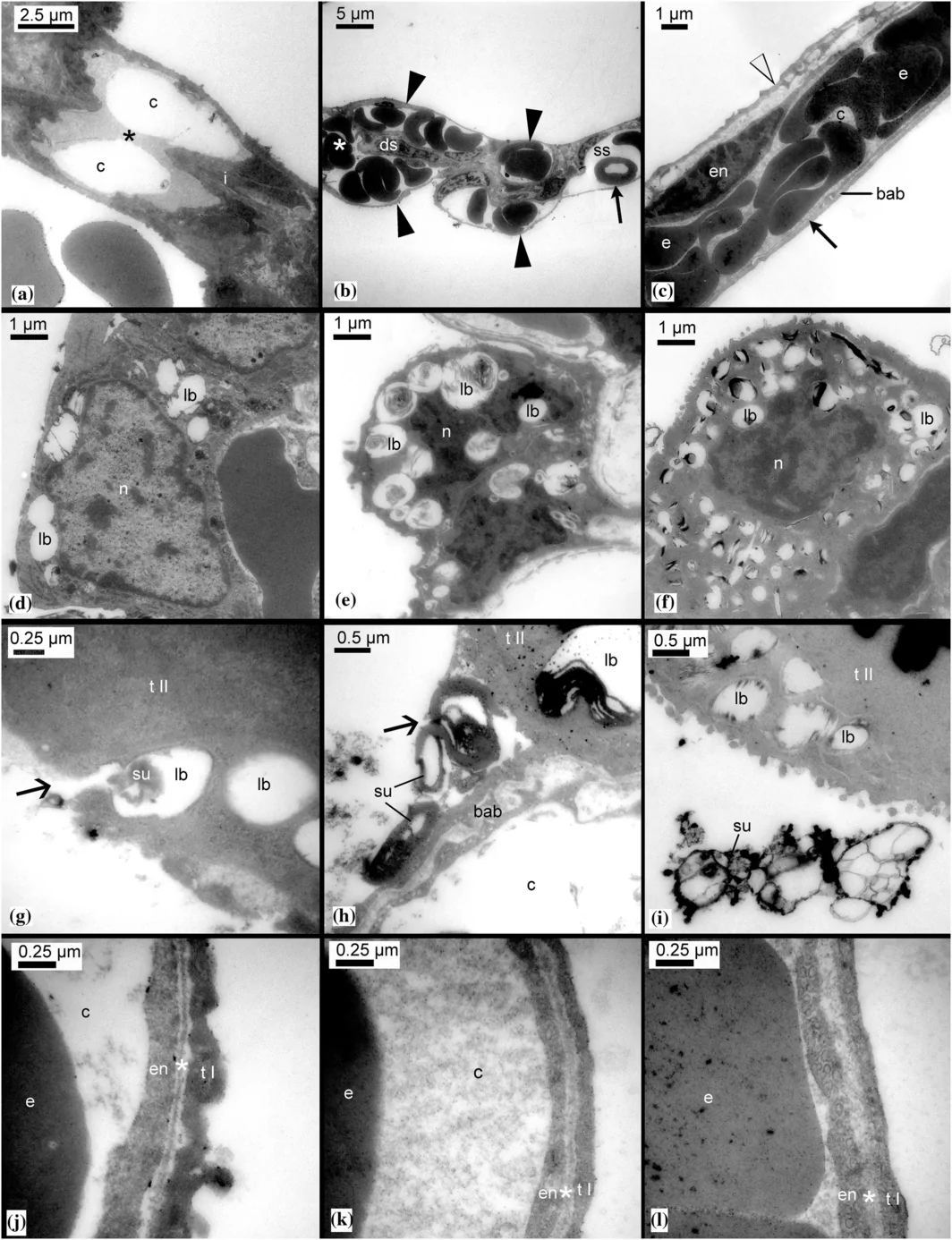
High magnification TEM micrographs of the interalveolar septum from the *Monodelphis lung* by 28 days pp (a), 35 days pp (b), and in adults (c) showing the steps in septal maturation, a process which entails interstitial tissue reduction and reorganization of the remaining septal components. During the process of microvascular maturation at 28 days pp, the two layers of the double capillary system come to lie close together as the interstitial tissue is reduced and finally fuse (indicated by asterisk). Further reduction of the interstitial tissue results in a few portions of the septum having a double capillary layer (indicated by arrowheads) while the majority consists mainly of a single capillary system (indicated by arrow). I adults the septa are mature, a single capillary system prevails and the interstitial tissue (indicated by open arrowhead) is scanty. Structural differences in the type II epithelial cells cannot be detected at the different ages (e.g., neonate (d), 14 days pp (e), 35 days pp (f)). Type II epithelial cells have a cuboidal shape and contain lamellar bodies, in which surfactant is produced. The lamellar bodies with mature surfactant release surfactant into the airspaces by means of exocytosis (g, indicated by arrow), the surfactant uncoils and covers the airspace surfaces to stabilize the airspaces (h, i). The blood‐air barrier has the same structure in all developmental stages examined (e.g., neonate (j), 35 days pp (k), adult (l)). It consists of capillary endothelial cells, type I epithelial cells and a basal lamina consisting of fused cells of both types (asterisk). days pp, days postpartum; bab, blood‐air barrier; c, capillary; ds, double capillary septum; e, erythrocyte; en, endothelium; I, interstitium; lb, lamellar body; n, nucleus; ss, single capillary septum; su, surfactant; t I, type I epithelial cell; t II, type II epithelial cell.

### 3.2 Lung morphometry

#### 3.2.1 Volume and volume proportions

The volume densities of the various components of the lung analyzed at the ultrastructural level are presented in Tables 1 and 2. Body mass, lung volumes, and proportions of the parenchymal and non‐parenchymal tissues of the lung are reported and discussed in a sister report, addressing morphological changes in the lung at light microscopic level (Ferner, 2025b). The values of the animals used for ultrastructural analysis are included here since they are requisite for calculation of most of the parameters in the current study.

**TABLE 1:** Body mass, lung volume, and volume densities of the various components of the lung parenchyma estimated for the various age groups of the gray short‐tailed opossum.

Age, days pp	Body mass, g	V(L), cm^3^	V(L), cm^3^ kg^−1^	Vv(p,L)	Vv(a,p)	Vv(s,p)	Vv(c,s)	Vv(e,s)
Neonate	0.125 (±0.002)	0.002 (±0.0002)	18.829 (±1.758)	0.887 (±0.012)	0.570^a^ (±0.043)	0.430^a^ (±0.043)	0.149 (±0.013)	0.083 (±0.011)
4	0.210 (±0.002)	0.004 (±0.0002)	21.038 (±1.112)	0.880 (±0.015)	0.482^a,b,c^ (±0.068)	0.518^a,b,c^ (±0.068)	0.179 (±0.028)	0.081 (±0.003)
7	0.270 (±0.016)	0.007 (±0.0002)	23.940 (±10.188)	0.833 (±0.009)	0.450^a,b,c^ (±0.050)	0.550^a,b,c^ (±0.060)	0.148 (±0.002)	0.056 (±0.007)
11	0.536 (±0.079)	0.015 (±0.002)	28.203 (±1.503)	0.855 (±0.008)	0.440^a,b,c^ (±0.059)	0.560^a,b,c^ (±0.059)	0.141 (±0.004)	0.054 (±0.006)
14	1.007 (±0.002)	0.030 (±0.001)	29.938 (±1.116)	0.791 (±0.011)	0.340^c,d^ (±0.003)	0.660^c,d^ (±0.003)	0.140 (±0.009)	0.065 (±0.006)
21	2.300 (±0.019)	0.075 (±0.007)	32.548 (±2.969)	0.839 (±0.005)	0.419^a,b,c^ (±0.021)	0.581^a,b,c^ (±0.021)	0.121 (±0.010)	0.053 (±0.005)
28	4.870 (±0.119)	0.202 (±0.009)	41.424 (±1.733)	0.777 (±0.029)	0.536^a,b,d^ (±0.030)	0.464^a,b,d^ (±0.030)	0.161 (±0.009)	0.052 (±0.010)
35	6.200 (±0.671)	0.370 (±0.040)	59.700 (±1.322)	0.777 (±0.006)	0.575^a,b,d^ (±0.023)	0.425^a,b,d^ (±0.023)	0.193 (±0.013)	0.060 (±0.007)
49 pp	12.107 (±0.637)	0.466 (±0.028)	38.501 (±0.758)	0.799 (±0.027)	0.578^a,b,d^ (±0.047)	0.422^a,b,d^ (±0.047)	0.144 (±0.019)	0.092 (±0.016)
57	19.057 (±2.137)	0.740 (±0.070)	39.141 (±1.431)	0.765 (±0.039)	0.627^a,b^ (±0.031)	0.373^a,b^ (±0.031)	0.173 (±0.020)	0.079 (±0.005)
Adult	74.740 (±4.902)	2.218 (±0.275)	29.606 (±2.803)	0.812 (±0.013)	0.757^a,b^ (±0.042)	0.243^a,b^ (±0.042)	0.138 (±0.030)	0.048 (±0.009)

*Note*: The body mass, the lung volume V(L), and the parenchymal proportion Vv(p,L) were estimated in a previous study (Ferner, 2025a, 2025b) and are included here since estimation of absolute and specific values of the other components is based on these values. The densities of air spaces Vv(a,p), the entire septum Vv(s,p), the capillary lumina Vv(c,s), and erythrocytes Vv(e,s) are presented. In this and subsequent tables, values are given as mean ± SEM. Values significantly different (*p* ≤ 0.05, ANOVA, Tukey) from each other are indicated by different letters. If values have one letter in common, they are not significantly different from each other. Values of Vv(c,s) and Vv(e,s) have no significant differences. The number of individuals is 3 per age group. days pp, days postpartum. The volume densities and volumes of non‐parenchyma Vv(np,L), V(np) and its components bronchioles Vv(b,np), blood vessels >30 μm Vv(v,nb) and connective tissue Vv(t,np) have been published previously in Ferner (2025b).

**TABLE 2:** Volume densities of the septal components of the lung parenchymal tissue estimated for the various age groups of *Monodelphis domestica*.

Age, days pp	Vv(st,s)	Vv(ep1,s	Vv(ep2,s)	Vv(i,s)	Vv(en,s)	Vv(lb,ep2)
Neonate	0.281 ± 0.051^a,b,c,d^	0.038 ± 0.003	0.032 ± 0.003	0.179 ± 0.046^a,b,c^	0.031 ± 0.002	0.185 ± 0.011^a^
4	0.340 ± 0.056^a,b,c,d^	0.054 ± 0.011	0.043 ± 0.009	0.199 ± 0.036^a,b,c^	0.044 ± 0.004	0.204 ± 0.016^a,b^
7	0.402 ± 0.049^b,c,d^	0.060 ± 0.005	0.054 ± 0.007	0.224 ± 0.038^b,c^	0.063 ± 0.006	0.261 ± 0.028^a,c^
11	0.418 ± 0.060^b,c,d^	0.052 ± 0.008	0.052 ± 0.008	0.262 ± 0.004^b,c^	0.052 ± 0.002	0.236 ± 0.024^a,c^
14	0.521 ± 0.009^d^	0.078 ± 0.002	0.070 ± 0.005	0.326 ± 0.007^c^	0.046 ± 0.002	0.335 ± 0.017^b,c,d^
21	0.459 ± 0.016^c,d^	0.081 ± 0.007	0.081 ± 0.007	0.232 ± 0.036^b,c^	0.065 ± 0.008	0.319 ± 0.007^c,d^
28	0.315 ± 0.033^b,c^	0.064 ± 0.007	0.045 ± 0.008	0.142 ± 0.031^a,b^	0.065 ± 0.008	0.307 ± 0.024^a,d^
35	0.230 ± 0.027^a,b^	0.065 ± 0.015	0.033 ± 0.009	0.081 ± 0.011^b^	0.051 ± 0.004	0.325 ± 0.013^c,d^
49	0.278 ± 0.029^b^	0.098 ± 0.013	0.041 ± 0.014	0.099 ± 0.014^b^	0.040 ± 0.001	0.314 ± 0.033^a,d^
57	0.199 ± 0.013^a,b^	0.076 ± 0.010	0.034 ± 0.005	0.049 ± 0.006^a,b^	0.040 ± 0.000	0.270 ± 0.009^b,c,d^
Adult	0.105 ± 0.015^a^	0.037 ± 0.006	0.016 ± 0.004	0.020 ± 0.003^a^	0.032 ± 0.005	0.264 ± 0.023^a,c^

*Note*: The densities of the septal tissue Vv(st,s), type I cells Vv(ep1,s), type II cells Vv(ep2,s), interstitium Vv(I,s), the endothelium Vv(en,s), and the lamellar bodies Vv(lb,ep2) within type II cells, are presented. Vv(st,s) is the summation of Vv(ep1,s), Vv(ep2,s), Vv(I,s), and Vv(en,s). Values are given as means ± SEM. Values significantly different (*p* ≤ 0.05, ANOVA, Tukey) from each other are indicated by different letters. If values have one letter in common, they are not significantly different from each other. Values of Vv(ep1,s), Vv(ep2,s), and Vv(en,s) have no significant differences. The number of individuals is 3 per age group. days pp, days postpartum.

The values for the proportions of the airspace volume Vv(a,p) are significantly higher than the proportions of the septal volume Vv(s,p) in the neonate and by 57 days pp in adults. During the saccular period from 4 to 21 days pp, Vv(s,p) is higher than Vv(a,p). From day 28, at the time alveolarization begins, the proportions reverse and Vv(a,p) is higher than Vv(s,p) until adulthood.

The volumes of the parenchymal airspaces V(a) and total septal tissue V(s) increase proportional to body mass (Table 3). The value of V(a) changes from a low of 1.188 ± 0.155 mm^3^ in the neonate to 1372.141 ± 191.692 mm^3^ in the adults, a 1150‐fold increase. V(s) changes from a low of 0.881 ± 0.089 mm^3^ in neonates to a high of 433.643 ± 93.312 mm^3^ in adults, a 492‐fold increase.

**TABLE 3:** Volumes of the various components of the lung parenchyma estimated for the various age groups.

Age, days pp	V(a), mm^3^	V(c), mm^3^	Vs, mm^3^	V(st), mm^3^	V(i), mm^3^	V(ep1), mm^3^	V(ep2)I, mm^3^	V(en), mm^3^	V(lb), mm^3^
Neonate	1.188 (±0.155)	0.304 (±0.021)	0.881 (±0.089)	0.577 (±0.108)	0.370 (±0.097)	0.078 (±0.008)	0.066 (±0.002)	0.064 (±0.002)	0.012 (±0.001)
4	1.900 (±0.288)	0.729 (±0.141)	2.114 (±0.418)	1.385 (±0.278)	0.782 (±0.188)	0.213 (±0.054)	0.170 (±0.045)	0.168 (±0.018)	0.036 (±0.012)
7	2.641 (±0.300)	0.904 (±0.173)	3.353 (±0.552)	2.450 (±0.039)	1.373 (±0.283)	0.360 (±0.032)	0.330 (±0.055)	0.387 (±0.050)	0.091 (±0.023)
11	5.328 (±0.126)	1.811 (±0.258)	7.538 (±1.954)	5.727 (±1.703)	3.614 (±1.137)	0.718 (±0.224)	0.712 (±0.212)	0.683 (±0.131)	0.182 (±0.071)
14	8.411 (±0.303)	3.513 (±0.353)	16.353 (±0.581)	12.840 (±0.229)	8.032 (±0.118)	1.946 (±0.111)	1.709 (±0.069)	1.153 (±0.079)	0.581 (±0.049)
21	26.070 (±1.890)	7.727 (±1.250)	36.572 (±3.977)	28.844 (±2.768)	14.140 (±1.895)	5.218 (±0.891)	5.272 (±1.542)	4.215 (±0.858)	1.659 (±0.460)
28	84.138 (±7.369)	25.209 (±1.784)	74.292 (±4.149)	49.083 (±4.902)	21.931 (±4.373)	9.896 (±1.038)	7.116 (±1.433)	10.140 (±1.447)	2.258 (±0.533)
35	162.75 (±9.662)	55.396 (±6.764)	123.904 (±19,59)	68.016 (±1.403)	23.297 (±3.805)	19.870 (±5.986)	10.281 (±3.779)	14.569 (±1.883)	3.226 (±1.072)
49	228.906 (±19.57)	53.874 (±8.627)	167.879 (±20.59)	103.509 (±1.325)	36.719 (±3.818)	36.475 (±5.112)	15.473 (±5.580)	14.841 (±0.117)	5.340 (±2.414)
57	348.000 (±3.884)	98.038 (±1.528)	210.049 (±26.085)	112.011 (±1.136)	26.962 (±0.297)	43.306 (±7.245)	19.166 (±3.759)	44.697 (±0.476)	5.079 (±0.821)
Adult	1372.141 (±191.692)	323.890 (±49.211)	433.643 (±93.312)	212.516 (±28.464)	36.226 (±7.202)	67.271 (±12.931)	39.003 (±6.557)	70.016 (±9.372)	7.128 (±0.981)

*Note*: The volumes of the septal tissue V(st), type I cells V(ep1), type II cells V(ep2), interstitium V(i), the endothelium V(en), and the lamellar bodies V(lb) within type II cells, are presented. Values are given as means ± SEM The number of individuals is 3 per age group. days pp, days postpartum.

The volume densities of the pulmonary capillary blood Vv(c,s) and capillary erythrocytes Vv(e,s) show no significant changes in the various developmental stages (Table 1). During the entire postnatal growth period, the volume density of the capillary endothelium Vv(en,s) does not change significantly (Table 2). On regression analysis, both the volume of the capillary endothelium V(en) and the volume of the pulmonary capillary blood V(c) show a strong positive correlation with body mass (Figure 5).

**FIGURE 5:**
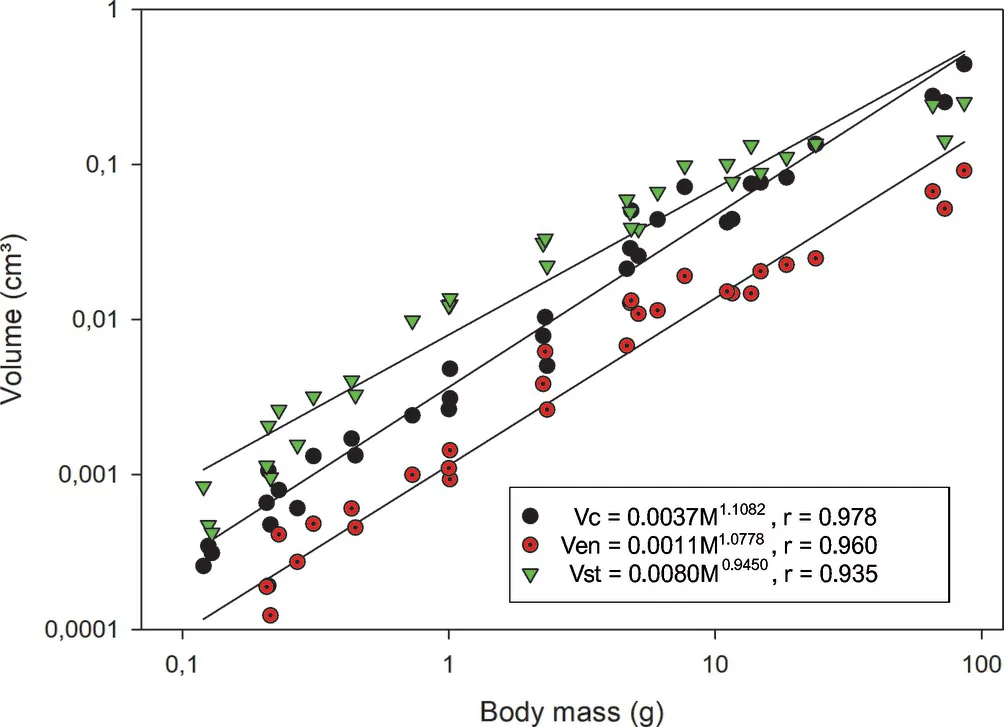
Double logarithmic plots of the volumes of the septal tissue V(st), the capillary endothelium V(en), and the capillary blood V(c) against body mass for *Monodelphis domestica* of various ages. The volume of the septal tissue increases with body weight until 57 days pp. In adults, no further increase of septal tissue volume can be observed, since interstitial tissue is greatly reduced and only alveolar septa with large central capillaries can be found. The volumes of capillaries and erythrocytes show a steady increase with body weight. In this and subsequent figures, the graphs are based on individual animal data, and respective allometric equations are provided, where M refers to body mass in grams.

The proportion of the solid component of the septal tissue Vv(st,s) increases from 0.281 ± 0.051 in neonates to a maximum of 0.521 ± 0.009 by 14 days pp. After that time Vv(st,s) decreases to 0.105 ± 0.015 in adults. The proportions of Vv(st,s) during mid saccular stage (14–21 days pp) are significantly higher than the proportions found in animals at the alveolar stage (35 days pp and older). V(st) is positively correlated with body mass and increases virtually in direct proportion with body mass (*r* = 0.935) until the alveolar period, where V(st) remarkably decreases (Figure 5).

The individual solid components of the septal tissue [the interstitium, the squamous (type I cells) and cuboidal (type II cells) epithelia and the capillary endothelium] all show contrasting changes in the postnatal period. The proportion of type I pneumocytes Vv(ep1,s) is highest by 49 days pp (0.098 ± 0.013) and lowest in the neonate (0.038 ± 0.003) and adult (0.037 ± 0.006). Vv(ep2,s) varies widely across postnatal development being highest in the late saccular stage by 21 days pp (0.081 ± 0.007) and lowest in the adult (0.016 ± 0.004). However, significant differences in Vv(ep1,s) and Vv(ep2,s) cannot be detected (Table 2). Both the volumes of type I cells V(ep1) and type II cells V(ep2) are positively correlated with body mass (Figure 6). V(ep1) increases from 0.078 ± 0.008 mm^3^ in neonates to 67.271 ± 12.931 mm^3^ in adults, an 860‐fold increase. V(ep2) increases 590‐fold from 0.066 ± 0.002 mm^3^ to 39.003 ± 6.557 mm^3^ in the same time period.

**FIGURE 6:**
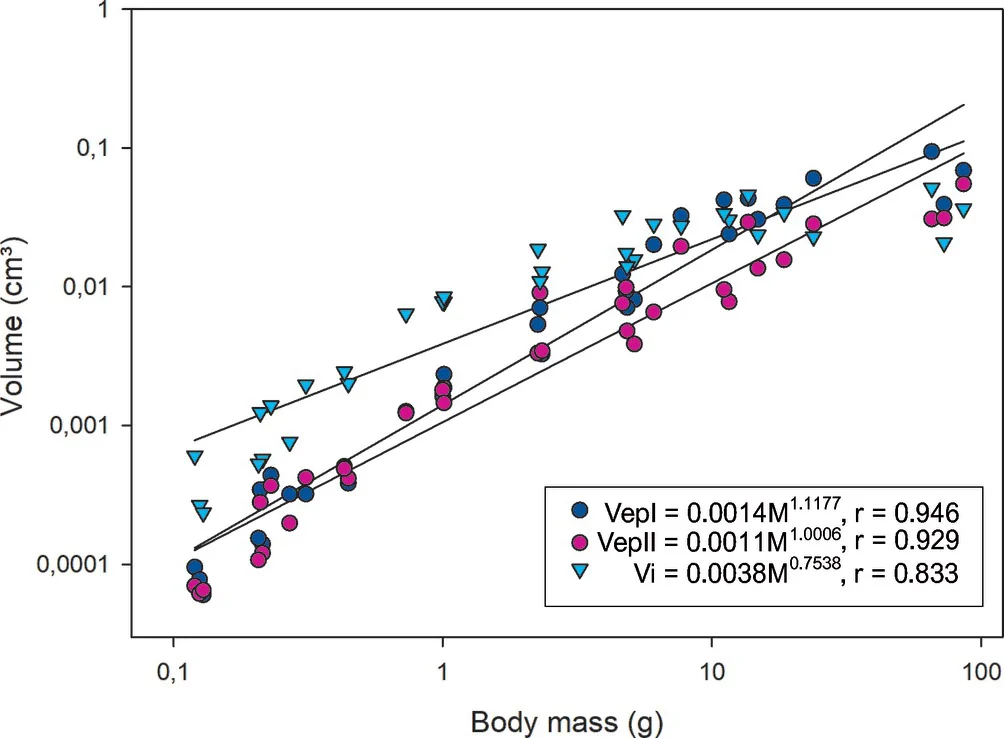
Double logarithmic plots of the volumes of type I epithelium V(ep1), type II epithelium V(ep2), and the interstitium V(i) against body mass for *Monodelphis domestica* of various ages. The volumes of the type I and type II epithelium septal tissue increase steadily with body weight from neonate to adult. The volume of the interstitium increases with body weight from neonate to 35 days pp, the time alveolarization starts. From this time on, no increase with body weight can be seen anymore. This reflects the continuous reduction of interstitial tissue during the alveolar period.

The volume density of the interstitial tissue increases from 0.179 ± 0.046 during the early saccular stage to a maximum of 0.326 ± 0.007 by 14 days pp which is significantly greater than Vv(,s) at 28 days pp and all the other subsequent stages. From postnatal day 21 Vv(,s) decreases to 0.020 ± 0.003 in the adults (Table 2). The volume of the septal interstitium V(i) shows diverse changes during the postnatal period and the correlation with body weight was weakest compared to all other septal components (Figure 6; *r* = 0.833). V(i) increases from neonate (0.370 ± 0.097 mm^3^) to 35 days pp (23.297 ± 3.805 mm^3^). With the beginning of the alveolar stage V(i) no longer increases proportionally with body mass, but remains stable at values between 26.962 ± 0.297 mm^3^ and 36.719 ± 3.818 mm^3^.

Type II epithelial cells have a cuboidal shape and contain lamellar bodies, in which surfactant is produced (Figure 4d–i). The presence of lamellar bodies, cell components located within the type II epithelial cells and the location of surfactant synthesis, is an indicator of surfactant production. The lamellar bodies with mature surfactant are triggered by intracellular pathways to traffic toward the apical plasma membrane, moving along microtubules and actin filaments, and dock at the plasma membrane. The lamellar body membrane fuses with the plasma membrane and releases surfactant into the airspaces (Figure 4g). Once secreted, the lamellar body content expands into tubular myelin and spreads to form a surfactant film at the air‐liquid interface in order to stabilize the airspaces (Figure 4h,i). The volume density of lamellar bodies Vv(lb,ep2) within type II epithelial cells is lowest in the neonate (0.185 ± 0.011; Table 2). Vv(lb,ep2) increases during the early saccular stage and reaches a maximum of 0.335 ± 0.017 by 14 days pp. During the late saccular and early alveolar stage, Vv(lb,ep2) remains high. After 35 days pp, Vv(lb,ep2) decreases to 0.264 ± 0.023 in adults. The total volume of lamellar bodies depends on the volume of type II epithelial cells. Following the multistage stratified sampling system, V(lb) results from multiplying Vv(lb,ep2) with Vv(ep2,s), Vv(s,p), Vv(p,l), and finally V(L). V(lb) increases in the postnatal period and is strongly correlated with body mass (Figure 7; *r* = 0.878).

**FIGURE 7:**
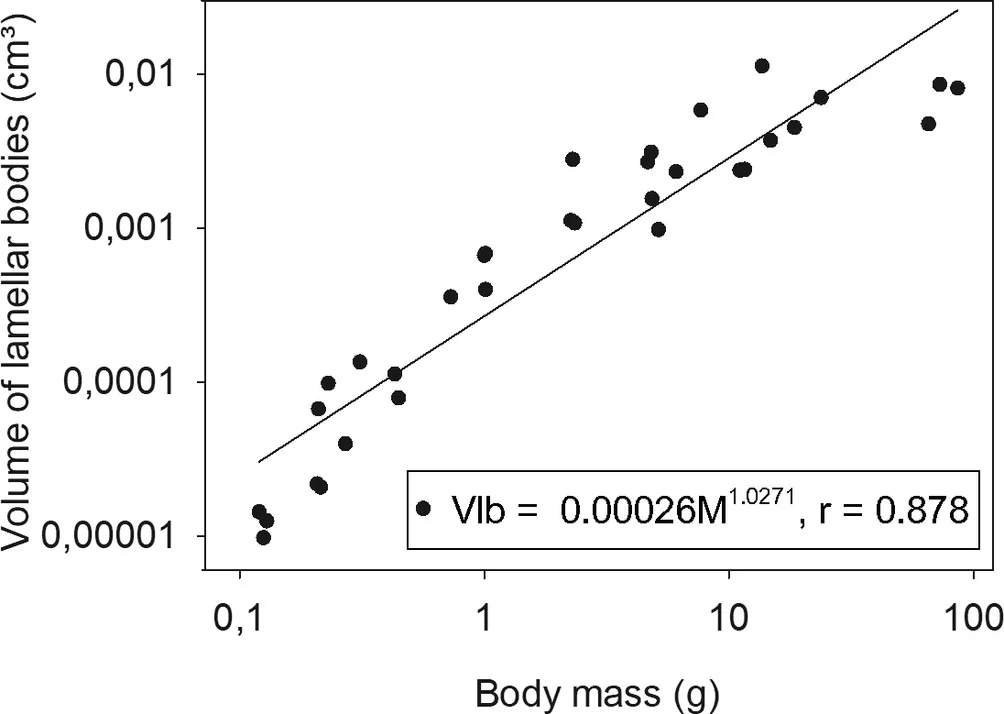
Double logarithmic plot of the volume of lamellar bodies V(lb) against body mass for *Monodelphis domestica* of various ages. The volume of the lamellar bodies increases with body weight until 57 days pp. In adults no further increase of t II epithelium volume can be observed.

#### 3.2.2 Surface densities and surface areas

Surface densities, absolute and body mass‐normalized surface areas for the air spaces and capillaries are provided in Table 4. In all developmental stages, surface densities of the capillaries Sv(c) very closely approximated the surface density of air spaces Sv(a). The surface density of the airspaces increased 2.5 times from neonate (147.1 ± 10.0 cm^−1^) to 35 days pp (372.4 ± 57.3 cm^−1^), the time when the alveolar stage is attained. During the alveolar period, Sv(a) remains on a similar level. In comparison, Sv(c) doubles in the same duration (0–35 days pp). Body mass‐normalized values for S(a) and S(c) appear to reach their peak in the late saccular/early alveolar stage by 35 days pp (Table 4). Both the airspace surface area S(a) and capillary surface area S(c) are positively correlated with body mass (Figure 8). Absolute surface area of the airspaces and capillaries increase 2000‐fold from values of 0.30 ± 0.02 cm^2^ (S(a)) and 0.41 ± 0.02 cm^2^ (S(c)) in the neonate to 681.81 ± 55.24 cm^2^ (S(a)) and 808.71 ± 12.07 cm^2^ (S(c)) in the adults.

**TABLE 4:** Surface densities, absolute and body mass‐normalized surface areas for the various age groups.

Age, days pp	Sv(a), cm^−1^	Sv(c), cm^−1^	S(a), cm^2^	S(c), cm^2^	S(a)/W, m^2^ kg^−1^	S(c)/W, m^2^ kg^−1^
Neonate	147.1 ± 10.0^a^	219.8 ± 13.8^a^	0.30 ± 0.02	0.41 ± 0.02	0.243 ± 0.19^a^	0.325 ± 0.02^a^
4	195.5 ± 5.6^a,b^	222.3 ± 20.3^a^	0.77 ± 0.07	0.86 ± 0.14	0.364 ± 0.33^a,b^	0.408 ± 0.05^a^
7	210.6 ± 12.6^a,c^	301.3 ± 22.6^a,b^	1.26 ± 0.07	1.80 ± 0.15	0.478 ± 0.54^a,b,d^	0.688 ± 0.11^a,b^
11	208.7 ± 39.2^a,c^	319.4 ± 46.8^a,b,c^	2.67 ± 0.53	3.86 ± 0.24	0.515 ± 0.12^a,b,c,d^	0.772 ± 0.13^a,b,c^
14	230.4 ± 8.4^b,c^	345.1 ± 4.6^b^	5.73 ± 0.34	8.55 ± 0.33	0.569 ± 0.33^b,e,d^	0.849 ± 0.32^b^
21	272.7 ± 14.4^b,c^	353.2 ± 1.9^b^	17.33 ± 2.37	22.19 ± 2.07	0.755 ± 0.11 ^e,f^	0.966 ± 0.09^b,d^
28	365.1 ± 22.9^c,e^	459.5 ± 10.5^c,d^	56.88 ± 3.39	71.97 ± 4.56	1.166 ± 0.05^c,f^	1.484 ± 0.12^b,e^
35	372.4 ± 57.3^c,f^	410.4 ± 10.90^b,d^	104.77 ± 14.79	117.17 ± 10.36	1.716 ± 0.24^c,e,f^	1.902 ± 0.39 ^e^
49	421.7 ± 6.64^d,e,f^	506.6 ± 26.85^c,d^	160.35 ± 2.75	187.07 ± 6.73	1.336 ± 0.08 ^f^	1.564 ± 0.13^c,d,e^
57	476.7 ± 32.45^d,e,f^	507.1 ± 28.1^c,d^	268.16 ± 29.38	284.82 ± 27.51	1.411 ± 0.40 ^f^	1.506 ± 0.05^d,e^
Adult	365.8 ± 29.15^c,f^	474.7 ± 71.1^b,d^	681.81 ± 55.24	808.71 ± 12.07	0.915 ± 0.62^d,f^	1.095 ± 0.07^b,d^

*Note*: The abbreviations Sv(a) and Sv(c) refer to the airspace and capillary surface area densities, while S(a) and S(c) represent the respective absolute values. The respective specific values are represented by the abbreviations S(a)/W and S(c)/W. Values are given as means ± SEM. Values significantly different (*p* ≤ 0.05, ANOVA, Tukey) from each other are indicated by different letters. If values have one letter in common, they are not significantly different from each other. The number of individuals is 3 per age group. days pp, days postpartum.

**FIGURE 8:**
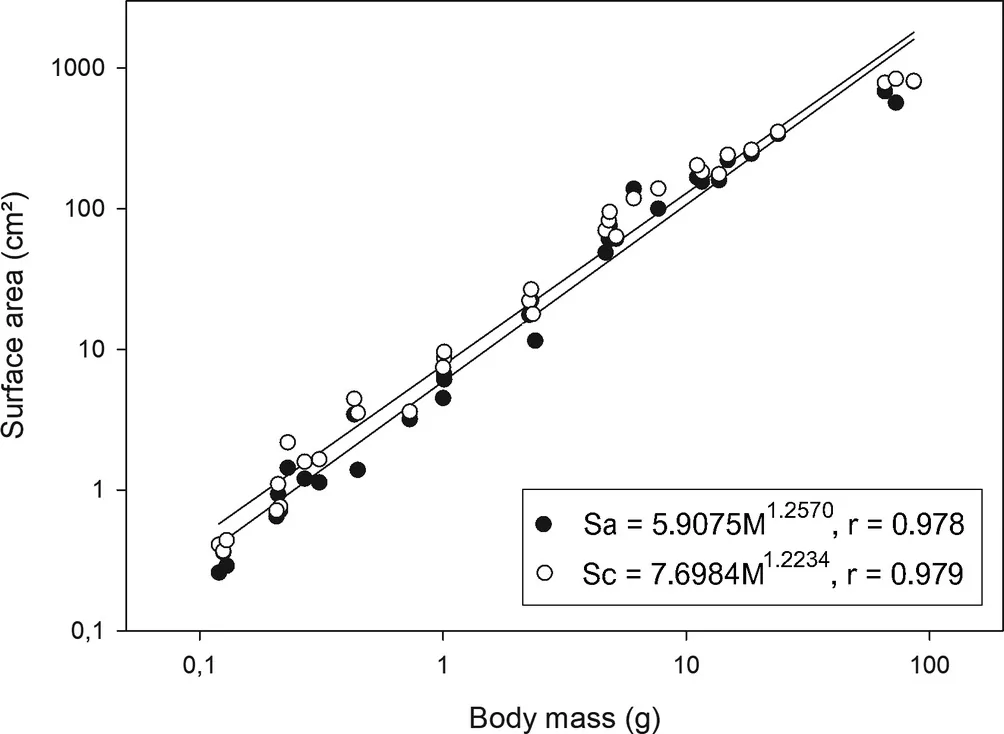
Bilogarithmic plots of the surface area of the airspaces (Sa) and that of the capillaries (Sc) against body mass.

#### 3.2.3 Barrier thickness

The blood‐air barrier of the lung parenchymal septa of the gray short‐tailed opossum has a similar structure in all developmental stages investigated (Figure 4j–l). It consists of a thin layer of type I epithelial cells, a layer of capillary endothelial cells, and a fused basal lamina consisting of both cell types. Estimates of the arithmetic mean thickness *τ*_t_, the measured barrier thickness [equals harmonic mean thickness (*τ*_hb_), the hematocrit (Hct), the septal thickness (2V(s)/S(a)), capillary loading (V(c)/S(a)), surface to volume ratio (S(a)/V(a))] in the various developmental stages are presented in Table 5. The measured barrier thickness of the blood‐air barrier is, with 0.43 ± 0.32 μm, highest in the neonate and becomes significantly thinner with the beginning of the alveolar stage, finally reaching a value of 0.24 ± 0.02 μm in adults. The arithmetic mean thickness τ_t_ is significantly higher in the mid and late saccular stages (11–21 days pp) than in adults.

**TABLE 5:** A tabulation of the arithmetic mean barrier thickness (*τ*_t_), the measured mean barrier thickness, the hematocrit (Hct), estimate of septal thickness (2V(s)/S(a)), capillary loading (V(c)/S(a)), and surface to volume ratio (S(a)/V(a)) for the various age groups.

Age, days pp	*τ*_τ_ (μm)	Measured barrier thickness (μm)	V(c)/S(a) (×10^−4^) (cm^3^ cm^−2^)	2V(s)/S(a) (×10^−4^) (cm^3^ cm^−2^)	S(a)/V(a) (cm^3^ cm^−2^)	Hct
Neonate	2.00 ± 0.51^a,b,c^	0.43 ± 0.32^a,c^	1.01 ± 0.03^a^	6.02 ± 1.00	259.4 ± 13.4^a^	0.63 ± 0.08
4	1.77 ± 0.21^a,c^	0.34 ± 0.50^a,b,c^	0.93 ± 0.12^a,b^	5.40 ± 0.63	461.9 ± 128.7^a,b^	0.48 ± 0.05
7	1.98 ± 0.37^a,b,c^	0.36 ± 0.01^a^	0.74 ± 0.17^a,b^	5.43 ± 1.07	490.9 ± 49.5^a,c^	0.39 ± 0.03
11	2.22 ± 0.46^a,b,c^	0.36 ± 0.01^a^	0.74 ± 0.11^a,b^	5.90 ± 1.08	496.97 ± 93.23^a,c^	0.38 ± 0.05
14	2.29 ± 0.19^a^	0.29 ± 0.01^c,d,e^	0.61 ± 0.05^b,c^	5.80 ± 0.37	677.30 ± 29.91^b,c,d^	0.46 ± 0.03
21	1.71 ± 0.08^a,d^	0.31 ± 0.01^a,d,f^	0.44 ± 0.01^b,c^	4.27 ± 0.15	659.33 ± 60.28^b,c,d^	0.45 ± 0.05
28	0.92 ± 0.14^c,d,e^	0.27 ± 0.01^c,f^	0.44 ± 0.01^b,c^	2.66 ± 0.29	682.53 ± 33.88^b,c,d^	0.33 ± 0.09
35	0.72 ± 0.14^c,f^	0.29 ± 0.00^c,f^	0.57 ± 0.10^a,c^	2.46 ± 0.42	645.95 ± 91.91^a,d^	0.31 ± 0.03
49	0.74 ± 0.08^c,f^	0.27 ± 0.02^c,f,g^	0.34 ± 0.06^b,c^	2.09 ± 0.25	715.74 ± 61.25^b,c,d^	0.64 ± 0.07
57	0.56 ± 0.02^b,e,f^	0.21 ± 0.01^b,g^	0.36 ± 0.02^b,c^	1.56 ± 0.03	773.69 ± 94.14^b,c,d^	0.47 ± 0.02
Adult	0.71 ± 0.02^b,e,f^	0.24 ± 0.02^b,e,f^	0.47 ± 0.04^b,c^	1.26 ± 0.21	515.13 ± 46.51^b,c,d^	0.25 ± 0.08

*Note*: Values are given as means ± SEM. Values significantly different (*p* ≤ 0.05, ANOVA, Tukey) from each other are indicated by different letters. If values have one letter in common, they are not significantly different from each other. Values of 2V(s)/S(a) and Hct have no significant differences. The number of individuals is 3 per age group. days pp, days postpartum.

Capillary loading (V(c)/S(a)) was highest in the neonate and significantly higher than in animals of 14 days and older, but was not significantly different among the subsequent groups. The septal tissue thickness (2V(s)/S(a)) in the canalicular and saccular stages tends to be higher than in animals at the alveolar stage; however, significant differences were not detected. S(a)/V(a) was significantly lower (*p* < 0.05) in the neonatal period than subsequent stages, reaching values above 600 cm^3^ cm^−2^ in the mid and late saccular and the alveolar stage (14–57 days pp) before decreasing slightly in the adult (Table 5).

## 4 DISCUSSION

Mammalian lung development seems to be highly conserved and follows similar developmental pathways in all mammalian species, including marsupials and monotremes (Ferner et al., 2009; Schittny, 2017; Szdzuy et al., 2008). The majority of studies focusing on mammalian lung development are performed in eutherian model species, and definitions for the different stages of lung development (e.g., canalicular, saccular, alveolar) are based on observations in altricial eutherian species, mostly during the prenatal period in a non‐ventilated state. Prolonged postnatal lung maturation in a ventilated and functioning state, characteristic for marsupials, may modify the lung developmental stages that were described in eutherians.

Due to their unique reproductive strategy, marsupials are born earlier in the developmental trajectory of lung development than eutherian mammals. In marsupials with more developed neonates, the prenatal establishment of a functional lung happens rapidly. In the bandicoot (*Isoodon macrourus*), for example, formation of a functional lung occurs in the last 3–4 days of gestation (Gemmell & Little, 1982), while in the tammar wallaby the transition from canalicular into saccular stage takes place in the last 3 days of gestation (Runciman et al., 1996). In less developed marsupial neonates, the saccular stage is attained in the early postnatal period, within the first postnatal days in the quokka (Burri et al., 2003; Makanya et al., 2001), by 5 days in the eastern quoll (*Dasyurus viverrinus*) (Ferner, 2021b), and between 4 and 7 days in the gray short‐tailed opossum (Ferner, 2025b).

From a short canalicular stage, the gray short‐tailed opossum lung enters a prolonged period of saccular stage. This time period is characterized by continued formation of transitory saccules, which are progressively subdivided by septation into new generations of saccules. The process of saccule multiplication is similar to alveolar formation, described by Burri et al. (1974). The main difference to alveolarization is that sacculation is not followed by microvascular maturation, the process that leads to the transformation from a double to a single capillary system. Sacculation is accompanied by tissue proliferation, which is indicated by high values of the estimator of septal tissue thickness 2V(s)/S(a) during the mid and late saccular stage (4–21 days pp). Similar observations were made in the developing lung of the quokka (Burri et al., 2003). A further support for tissue proliferation during the mid‐ and late‐ saccular stage is the fact that the volume densities of septal tissue Vv(st,s), interstitial tissue Vv(I,s), and type II epithelial cells Vv(ep2,s) are highest by 14 and 21 days pp (Table 2).

Vv(I,s) and Vv(ep2,s) decrease rapidly in the alveolar stage, reaching the lowest values in the adults. Reduction of type II epithelial cells is caused partly by apoptosis (Schittny et al., 1998) and partly due to conversion to type I epithelial cells (Kauffman et al., 1974; Mercurio & Rhodin, 1976). The amount of the interstitial tissue might be decreased by apoptosis of fibroblasts (e.g., degradation of components of the extracellular matrix, such as collagen, laminin, fibronectin, and elastin by matrix metalloproteinases), which lead to a reduction of the fibrous components and the amorphous ground substance of the interstitium (Burri et al., 2003; Mannello et al., 2005).

The proportion of capillary volume in the septa Vv(c,s) does not change significantly over the course of postnatal development in the gray short‐tailed opossum. This pattern is different from what has been observed in ultrastructural studies of lung development in the quokka and tammar wallaby. Runciman et al. (1999) and Burri et al. (2003) report highest values of Vv(c,s) in adults and lower values in younger animals. The high values of Vv(c,s) in adults and late saccular stages are discussed as indicators for septal maturation, involving tissue reduction of the interstitium and attenuation of the alveolar epithelium, thus leaving the capillaries to occupy a large portion of the septal parenchyma (Burri et al., 2003). The process of microvascular maturation takes place in the gray short‐tailed opossum by 28–35 postnatal days (Figure 4a–c). The double capillary system is reduced to a single capillary one mainly by fusion of portions of the two layers (Burri, 1974; Caduff et al., 1986).

Similarly to what is reported for the quokka (Burri et al., 2003) and tammar wallaby (Runciman et al., 1999), the volume proportion of capillary endothelium Vv(en,s) does not change significantly with age for the gray short‐tailed opossum.

In the current study both surface densities, that of the airspaces Sv(a) and that of the capillary Sv(c) are highest in the alveolar stage from 28 days pp to adult, reflecting the structural transformation of the lung parenchyma associated with alveolarization, leading to a marked increase in gas exchange surface area. The quokka similarly exhibits the highest values of Sv(a) and Sv(c) in the microvascular maturation stage, that is, after alveolarization (Burri et al., 2003).

The absolute surface areas S(a) and S(c) vary widely among stages. Absolute surface areas of the airspaces and of the capillaries increase 2000‐fold from neonate to adult stage. For the quokka a 1000‐fold increase in Sa and 800‐fold increase in Sc is reported from early saccular to adult stage (Burri et al., 2003). The body mass‐normalized airspace and capillary surface areas increase significantly to a value above 1 with beginning alveolarization. The higher values for the surface to volume ratio (S(a)/V(a)) of the airspaces observed in the late saccular, microvascular maturation and early alveolar stages is a reflection of the complexity of the parenchymal structure at these stages as a result of septation. Similarly to the findings reported for the quokka (Burri et al., 2003), the capillary loading V(c)/S(a) in the gray short‐tailed opossum is highest early in the saccular stage (4–11 days pp) and declines in the late saccular and alveolar stage. At the transition from the canalicular to the saccular stage by 4–7 days pp, the septa acquire a double capillary layer, which results in a high capillary volume. Subsequently, there is a fusion of the opposite capillaries to form a single capillary system. It is still unknown if capillaries are lost during this process (Burri et al., 2003). Septal maturation in adults entails a loss of the interstitium and probably subsequent expansion of the capillaries to fill the space left by the reduced interstitial tissue.

In the developing gray short‐tailed opossum, the arithmetic mean barrier thickness ranges between 0.56 and 2.22 μm, which is similar to values reported for adults of other marsupial species [quokka: 1.29 μm (Burri et al., 2003); kowari (*Dasyuroides byrnei*): 1.4 μm (Hallam et al., 1989); and the eutherian rat (*Rattus norvegicus*): 1.54 μm (Kalenga et al., 1995)]. The only other study describing ultrastructural changes during marsupial postnatal lung development, in the quokka, reports a higher arithmetic mean barrier thickness of 5.48–8.71 μm from the onset to late saccular stage (Burri et al., 2003).

The mean measured barrier thickness in the lung of the gray short‐tailed opossum is highest in the neonate (0.43 μm). During postnatal development the mean measured barrier thickness decreases continuously to 0.24 μm in adults. The harmonic mean barrier thickness reported for the developing quokka was higher (0.82–1.63 μm) than in the gray short‐tailed opossum (Burri et al., 2003). The harmonic mean barrier thickness of adult marsupials (quokka wallabies: 1.04 μm, tammar wallabies: 0.27 μm) and of the eutherian rat (0.64 μm) is in a similar range as that found in the gray short‐tailed opossum.

The parenchymal phenotypes during pre‐ and postnatal lung development are used to define the different stages of lung development (Burri, 1999; Schittny, 2017).

In the lung of the newborn *Monodelphis domestica*, the terminal air spaces are lined by stretches of cuboidal type II epithelial cells and squamous type I epithelial cells between portions of respiratory capillaries with a thin air‐blood barrier. This morphology is characteristic of the canalicular stage. Similar lining of the air spaces has been described in the canalicular lungs of the newborn northern and eastern quoll (Ferner, 2021b; Gemmell & Nelson, 1988) and the quokka (Makanya et al., 2007).

The effectiveness of ventilation is influenced by both neuronal control and the mechanical properties of the respiratory system. For proper lung function the surface tension at the lung surface/air interface must be overcome. Despite their immaturity, marsupial newborns show qualitative characteristics of mature lungs, such as a mature surfactant system (Makanya et al., 2007; Miller et al., 2001; Orgeig & Daniels, 2001; Ribbons et al., 1989) and a thin blood‐gas barrier (Runciman et al., 1996; Szdzuy et al., 2008). Pulmonary surfactant is a complex of lipids and protein (mainly phospholipids) secreted by the type II epithelial cells, stored in lamellar bodies and eventually released into the fluid lining the lung to reduce the surface tension associated with changes in lung surface area (Frappell & MacFarlane, 2006).

The lamellar bodies within the alveolar type II epithelial cells of *Monodelphis domestica* exhibit the characteristic structure found in other mammalian lungs throughout all stages of development. They normally have the onion‐like packing of biomembranes and also lattice‐like arranged tubular myelin is present (Szdzuy, 2006). The poor preservation of lipids in this study might result from the fixing agent (Karnovsky's fixative instead of 2.5% Glutaraldehyd). Karnovsky's fixative as initial fixing agent (postfixed with 2.5% Glutaraldehyd) has been used to allow a deeper tissue penetration to reach the lungs in the whole immersed torsos of the small neonates. However, Karnovsky's fixative might cause inappropriate lipid preservation, leading to the observed artifacts. Lamellar bodies have been reported in all marsupial newborns studied so far (Burri et al., 2003; Gemmell & Nelson, 1988; Krause & Leeson, 1975; Miller et al., 2001; Ribbons et al., 1989). The distribution and abundance of the phospholipid classes have been investigated only in the tammar wallaby and is comparable to that present in eutherians (Ribbons et al., 1989).

In the gray short‐tailed opossum, the volume density of lamellar bodies within the type II epithelial cells is highest from 14 to 49 days pp, indicating an accelerated surfactant production during the time of septal transformation and early alveolarization. This suggests that the mechanisms of storage of surfactant in lamellar bodies, exocytosis of active surfactant and formation and structure of surfactant films may remain consistent across developmental stages (Figure 4d–i).

From the quantitative data presented here, the successive events characterizing postnatal lung development in the gray short‐tailed opossum can be derived. After a brief canalicular stage, a longer saccular stage leads to a drastic increase in lung parenchyma. During this time period, tissue proliferation is intense with repetitive steps of septation, which increase the number of airspace generations. By 28 days pp the first generation of alveoli is formed and gas exchange surface area increases markedly. Alveolarization is mainly accompanied by redistribution of the tissues and septa become more elongated. During continued alveolarization, further remodeling takes place, which involves reduction in the volume of interstitium and conversion of the double capillary system into a single one, followed by the expansion of the airspaces.

## 5 CONCLUSION

Despite the immaturity of the gray short‐tailed opossum lung at birth, the lung is functioning and takes over the main part of respiration. However, cutaneous respiration may supplement pulmonary respiration during the first postnatal days (Ferner, 2025a, 2025b). The postnatal development of the septal components of the lung parenchyma in *Monodelphis domestica* is characterized by the formation of a double capillary system at the transition from the canalicular to the saccular stage, extensive tissue proliferation during the saccular stage, the transformation from double to single capillary septa during microvascular maturation at the transition to the alveolar stage and finally a reduction of interstitium and expansion of capillaries in the adults. This structural transformation is accompanied by an increase in gas exchange surface area and thinning of the diffusion barrier in order to meet the metabolic requirements of the developing young.

## AUTHOR CONTRIBUTIONS

**Kirsten Ferner:** Conceptualization; investigation; funding acquisition; writing – original draft; methodology; visualization; data curation; formal analysis.

## CONFLICT OF INTEREST STATEMENT

The author declares no conflicts of interest.

## Supporting information


**Table S1.** Age, body mass, lung volume (VL), surface area density (Sva), absolute surface area (Sa), weight specific surface area (Sa/W) and proportions of parenchyma (Vvp) and non‐parenchyma (Vvnp) and its components (Vva, airspaces; Vvs, septa; Vvb, airways; Vvv, blood vessels; Vvt, connective tissue) of all animals examined in this study.

## Data Availability

The data that support the findings of this study are available under https://doi.org/10.6084/m9.figshare.29460002 and original TEM images for this publication are published under https://doi.org/10.6084/m9.figshare.29460089, https://doi.org/10.6084/m9.figshare.29460254, and https://doi.org/10.6084/m9.figshare.29460398.
