# Supplementary material for: Morphometric analysis of postnatal lung development in the gray short‐tailed opossum (Monodelphis domestica): An ultrastructural study
Source: Anat Rec (Hoboken). 2025 Dec 22;309(11):3397–415. doi: 10.1002/ar.70122 (PMC13644897; doi:10.1002/ar.70122)
Supplement: Supplementary file 1 [file AR-309-3397-s001.docx]

Supplementary table – Age, Body mass, Lung volume (VL), Surface area density (Sva), absolute surface area (Sa), weight specific surface area (Sa/W) and proportions of parenchyma (Vvp) and non-parenchyma (Vvnp) and its compontents (Vva, airspaces; Vvs, septa; Vvb, airways; Vvv, blood vessels; Vvt, connective tissue) of all animals examined in this study

| Specimen | Age (days) | Body mass (g) | VL (cm³) | VL (cm³kg^-1^) | Sva (cm^-1^) | Sa (cm²) | Sa/W (m^2^kg^-1^) | Svc (cm^-1^) | Sc (cm²) | Sc/W (m^2^kg^-1^) | Vvp | Vva | Vvs | Vvc | Vve | Vvst | Vven | VvepI | VvepII | Vvi | Vvlb |
| --- | --- | --- | --- | --- | --- | --- | --- | --- | --- | --- | --- | --- | --- | --- | --- | --- | --- | --- | --- | --- | --- |
| 1965_1 | 0 | 0.12 | 0.00234 | 19.495 | 124.1 | 0.259 | 0.216 | 195.7 | 0.408 | 0.340 | 0.891 | 0.475 | 0.525 | 0.123 | 0.059 | 0.402 | 0.032 | 0.046 | 0.034 | 0.291 | 0.204 |
| 2350_7 | 0 | 0.125 | 0.00277 | 22.181 | 151.2 | 0.361 | 0.289 | 211.2 | 0.367 | 0.294 | 0.860 | 0.658 | 0.342 | 0.145 | 0.086 | 0.197 | 0.027 | 0.033 | 0.026 | 0.112 | 0.158 |
| 2350_3 | 0 | 0.129 | 0.00191 | 14.81 | 165.9 | 0.288 | 0.224 | 252.4 | 0.439 | 0.340 | 0.910 | 0.579 | 0.421 | 0.179 | 0.105 | 0.242 | 0.034 | 0.035 | 0.038 | 0.136 | 0.192 |
| N=3 |  | 0.125 ± 0.002 | 0.0023 ± 0.0002 | 18.829 ± 1.758 | 147.1 ± 9.99 | 0.303 ± 0.025 | 0.243 ± 0.019 | 219.8± 13.8 | 0.405± 0.017 | 0.325± 0.017 | 0.887 ± 0.012 | 0.570 ± 0.043 | 0.430 ± 0.043 | 0.149± 0.013 | 0.083± 0.011 | 0.281± 0.051 | 0.031± 0.002 | 0.038± 0.003 | 0.032± 0.003 | 0.179± 0.046 | 0.185± 0.011 |
| 1995_2 | 4 | 0.21 | 0.00498 | 23.74 | 204.4 | 0.934 | 0.445 | 254.4 | 1.099 | 0.523 | 0.867 | 0.279 | 0.721 | 0.245 | 0.085 | 0.476 | 0.044 | 0.080 | 0.065 | 0.288 | 0.239 |
| 2257_3 | 4 | 0.214 | 0.00415 | 19.37 | 200.2 | 0.717 | 0.335 | 212.4 | 0.760 | 0.355 | 0.864 | 0.600 | 0.400 | 0.133 | 0.073 | 0.267 | 0.034 | 0.039 | 0.034 | 0.160 | 0.171 |
| 2225_4 | 4 | 0.207 | 0.00414 | 20.01 | 182.0 | 0.649 | 0.314 | 200.2 | 0.714 | 0.345 | 0.861 | 0.567 | 0.433 | 0.158 | 0.084 | 0.275 | 0.053 | 0.043 | 0.030 | 0.149 | 0.202 |
| N=3 | 4 | 0.210 ± 0.002 | 0.00442 ± 0.00023 | 21.038 ± 1.112 | 195.5 ± 5.621 | 0.767 ± 0.070 | 0.364 ± 0.033 | 222.3± 20.33 | 0.858 ± 0.099 | 0.408 ± 0.047 | 0.864 ± 0.014 | 0.482 ± 0.068 | 0.518 ± 0.068 | 0.179 ± 0.028 | 0.081 ± 0.003 | 0.340 ± 0.056 | 0.044± 0.004 | 0.054± 0.011 | 0.043± 0.009 | 0.199± 0.036 | 0.204± 0.016 |
| 1987_2 | 7 | 0.310 | 0.00751 | 24.217 | 175.3 | 1.129 | 0.364 | 256.2 | 1.651 | 0.533 | 0.858 | 0.302 | 0.698 | 0.204 | 0.071 | 0.494 | 0.074 | 0.050 | 0.065 | 0.305 | 0.319 |
| 2383_5 | 7 | 0.230 | 0.00754 | 23.409 | 232.1 | 1.435 | 0.624 | 351.7 | 2.174 | 0.945 | 0.820 | 0.450 | 0.550 | 0.128 | 0.044 | 0.422 | 0.064 | 0.060 | 0.054 | 0.224 | 0.265 |
| 2383_4 | 7 | 0.270 | 0.00653 | 24.193 | 224.4 | 1.203 | 0.445 | 295.9 | 1.586 | 0.587 | 0.820 | 0.597 | 0.403 | 0.113 | 0.053 | 0.290 | 0.051 | 0.060 | 0.037 | 0.142 | 0.200 |
| N=3 |  | 0.270 ± 0.016 | 0.00719 ± 0.00023 | 23.940 ± 10.188 | 210.57 ± 12.58 | 1.255 ± 0.065 | 0.478 ± 0.054 | 301.3± 22.60 | 1.804 ± 0.152 | 0.688 ± 0.106 | 0.833 ± 0.009 | 0.450 ± 0.060 | 0.550 ± 0.060 | 0.148 ± 0.023 | 0.056± 0.007 | 0.402± 0.049 | 0.064± 0.006 | 0.060 ± 0.005 | 0.054 ± 0.007 | 0.224± 0.038 | 0.261 ± 0.028 |
| 1993_3 | 11 | 0.730 | 0.02056 | 28.161 | 182.0 | 3.179 | 0.435 | 206.1 | 3.599 | 0.493 | 0.850 | 0.297 | 0.703 | 0.138 | 0.042 | 0.565 | 0.057 | 0.072 | 0.070 | 0.366 | 0.290 |
| 2419_3 | 11 | 0.446 | 0.01117 | 25.036 | 142.1 | 1.386 | 0.311 | 361.8 | 3.529 | 0.791 | 0.873 | 0.529 | 0.471 | 0.136 | 0.068 | 0.335 | 0.047 | 0.039 | 0.043 | 0.207 | 0.189 |
| 2419_4 | 11 | 0.431 | 0.01354 | 31.411 | 302.0 | 3.437 | 0.797 | 390.4 | 4.443 | 1.031 | 0.841 | 0.495 | 0.505 | 0.150 | 0.051 | 0.355 | 0.053 | 0.045 | 0.043 | 0.214 | 0.230 |
| N=3 |  | 0.536 ± 0.079 | 0.01509 ± 0.00230 | 28.203 ± 1.503 | 208.69 ± 39.24 | 2.667 ± 0.527 | 0.510 ± 0.026 | 319.4± 46.77 | 3.857 ± 0.240 | 0.772 ± 0.127 | 0.855 ± 0.008 | 0.440 ± 0.059 | 0.560 ± 0.059 | 0.141 ± 0.004 | 0.054± 0.006 | 0.418 ± 0.060 | 0.052± 0.002 | 0.052 ± 0.008 | 0.052± 0.008 | 0.262± 0.042 | 0.236 ± 0.024 |
| 1994_4 | 14 | 1.01 | 0.02770 | 27.423 | 253.9 | 6.088 | 0.603 | 360.7 | 8.649 | 0.856 | 0.866 | 0.351 | 0.649 | 0.129 | 0.065 | 0.520 | 0.039 | 0.078 | 0.078 | 0.325 | 0.365 |
| 1994_5 | 14 | 1.00 | 0.02831 | 28.310 | 200.5 | 4.495 | 0.450 | 331.4 | 7.429 | 0.743 | 0.792 | 0.331 | 0.669 | 0.118 | 0.045 | 0.551 | 0.049 | 0.073 | 0.080 | 0.349 | 0.368 |
| 1994_6 | 14 | 1.01 | 0.03442 | 34.083 | 236.7 | 6.604 | 0.654 | 343.1 | 9.570 | 0.948 | 0.810 | 0.337 | 0.663 | 0.172 | 0.084 | 0.491 | 0.051 | 0.084 | 0.052 | 0.304 | 0.274 |
| N=3 |  | 1.007 ± 0.002 | 0.03014 ± 0.00115 | 29.938 ± 1.116 | 230.38 ± 8.412 | 5.729 ± 0.339 | 0.569 ± 0.033 | 345.1± 4.561 | 8.549 ± 0.331 | 0.849± 0.032 | 0.823 ± 0.012 | 0.340± 0.003 | 0.660 ± 0.003 | 0.140 ± 0.009 | 0.065 ± 0.006 | 0.521 ± 0.009 | 0.046± 0.002 | 0.078± 0.002 | 0.070 ± 0.005 | 0.326± 0.007 | 0.335± 0.017 |
| 2036 | 21 | 2.26 | 0.07571 | 33.499 | 280.2 | 1.553 | 0.777 | 352.9 | 22.107 | 0.978 | 0.827 | 0.378 | 0.622 | 0.125 | 0.042 | 0.497 | 0.061 | 0.085 | 0.053 | 0.298 | 0.336 |
| 2037 | 21 | 2.34 | 0.06044 | 25.829 | 239.1 | 12.204 | 0.522 | 349.4 | 17.830 | 0.762 | 0.844 | 0.467 | 0.533 | 0.098 | 0.055 | 0.434 | 0.051 | 0.064 | 0.068 | 0.252 | 0.312 |
| 2192 | 21 | 2.30 | 0.08813 | 38.315 | 298.8 | 22.244 | 0.934 | 357.6 | 26.618 | 1.157 | 0.845 | 0.412 | 0.588 | 0.139 | 0.063 | 0.448 | 0.084 | 0.095 | 0.122 | 0.148 | 0.308 |
| N=3 | 21 | 2.300 ± 0.019 | 0.07476 ± 0.00657 | 32.548 ± 2.969 | 272.73 ± 14.399 | 17.334 ± 2.368 | 0.755 ± 0.105 | 353.3± 1.944 | 22.189 ± 2.071 | 0.966± 0.093 | 0.839 ± 0.005 | 0.419 ± 0.021 | 0.581 ± 0.021 | 0.121 ± 0.010 | 0.053± 0.005 | 0.459 ± 0.016 | 0.065± 0.008 | 0.081± 0.008 | 0.081± 0.017 | 0.232± 0.004 | 0.319± 0.007 |
| 2058 | 28 | 4.66 | 0.18080 | 38.799 | 323.0 | 48.589 | 1.043 | 465.9 | 70.095 | 1.504 | 0.832 | 0.465 | 0.535 | 0.141 | 0.077 | 0.394 | 0.045 | 0.082 | 0.050 | 0.217 | 0.354 |
| 2043 | 28 | 5.15 | 0.20522 | 39.849 | 418.3 | 60.981 | 1.184 | 434.6 | 63.367 | 1.230 | 0.710 | 0.560 | 0.440 | 0.176 | 0.038 | 0.264 | 0.075 | 0.055 | 0.027 | 0.108 | 0.252 |
| 2193 | 28 | 4.80 | 0.219 | 45.625 | 354.2 | 61.081 | 1.273 | 478.0 | 82.441 | 1.718 | 0.787 | 0.585 | 0.415 | 0.167 | 0.040 | 0.287 | 0.074 | 0.054 | 0.057 | 0.101 | 0.314 |
| N=3 |  | 4.870 ± 0.119 | 0.20167 ± 0.00912 | 41.424 ± 1,733 | 365.138 ± 22.915 | 56.883 ± 3.386 | 1.166 ± 0.055 | 459.5 ± 10.578 | 71.968 ± 4.560 | 1.484 ± 0.115 | 0.777 ± 0.029 | 0.536 ± 0.030 | 0.464 ± 0.030 | 0.161 ± 0.009 | 0.052 ± 0.010 | 0.315 ± 0.033 | 0.065± 0.008 | 0.064± 0.007 | 0.045 ± 0.008 | 0.142± 0.031 | 0.307± 0.024 |
| 2194 | 35 | 7.68 | 0.465 | 60.547 | 281.2 | 99.824 | 1.2998 | 390.6 | 138.7 | 1.806 | 0.763 | 0.521 | 0.479 | 0.202 | 0.077 | 0.277 | 0.054 | 0.091 | 0.055 | 0.077 | 0.298 |
| 2404 | 35 | 4.840 | 0.300 | 61.983 | 325.5 | 76.168 | 1.5737 | 404.9 | 94.8 | 1.958 | 0.780 | 0.618 | 0.382 | 0.215 | 0.052 | 0.167 | 0.056 | 0.030 | 0.020 | 0.060 | 0.323 |
| 2405 | 35 | 6.081 | 0.344 | 56.570 | 510.5 | 138.3 | 2.275 | 435.8 | 118.1 | 1.942 | 0.788 | 0.586 | 0.414 | 0.163 | 0.051 | 0.245 | 0.042 | 0.074 | 0.024 | 0.105 | 0.353 |
| N=3 |  | 6.200 ± 0.671 | 0.36967 ± 0.04028 | 59.700 ± 1.322 | 372.40 ± 57.34 | 104.77 ± 14.79 | 1.716 ± 0.237 | 410.4 ± 10.90 | 117.17 ± 10.36 | 1.902 ± 0.039 | 0.777 ± 0.006 | 0.575 ± 0.023 | 0.425 ± 0.023 | 0.193 ± 0.013 | 0.051 ± 0.004 | 0.230 ± 0.027 | 0.051± 0.004 | 0.065± 0.015 | 0.033± 0.009 | 0.081± 0.011 | 0.325± 0.013 |
| 2049 | 49 | 13.64 | 0.534 | 39.159 | 406.0 | 159.01 | 1.166 | 448.8 | 175.8 | 1.289 | 0.733 | 0.469 | 0.531 | 0.191 | 0.125 | 0.339 | 0.037 | 0.111 | 0.074 | 0.117 | 0.387 |
| 2402 | 49 | 11.59 | 0.425 | 36.670 | 433.1 | 155.30 | 1.340 | 508.3 | 182.3 | 1.573 | 0.844 | 0.662 | 0.338 | 0.124 | 0.060 | 0.215 | 0.041 | 0.067 | 0.022 | 0.085 | 0.307 |
| 2403 | 49 | 11.09 | 0.440 | 39.675 | 426.2 | 166.73 | 1.503 | 562.7 | 203.1 | 1.832 | 0.820 | 0.604 | 0.394 | 0.117 | 0.092 | 0.279 | 0.042 | 0.117 | 0.026 | 0.094 | 0.249 |
| N=3 |  | 12.107 ± 0.637 | 0.46637 ± 0.02788 | 38.501 ± 0.758 | 421.731 ± 6.645 | 160.347 ± 2.749 | 1.336 ± 0.080 | 506.6 ± 26.85 | 187.07 ± 6.73 | 1.564 ± 0.013 | 0.799 ± 0.027 | 0.578 ± 0.047 | 0.422 ± 0.047 | 0.144 ± 0.019 | 0.092 ± 0.016 | 0.278 ± 0.029 | 0.040± 0.001 | 0.098± 0.013 | 0.041 ± 0.014 | 0.099± 0.008 | 0.314± 0.033 |
| 2195 | 57 | 23.83 | 0.910 | 38.187 | 555.1 | 338.7 | 1.121 | 575.5 | 351.2 | 1.474 | 0.671 | 0.555 | 0.445 | 0.222 | 0.092 | 0.224 | 0.041 | 0.099 | 0.046 | 0.038 | 0.249 |
| 2413 | 57 | 14.81 | 0.630 | 42.539 | 426.3 | 220.7 | 1.490 | 466.3 | 241.4 | 1.630 | 0.822 | 0.682 | 0.318 | 0.148 | 0.072 | 0.170 | 0.039 | 0.059 | 0.026 | 0.046 | 0.273 |
| 2416 | 57 | 18.53 | 0.680 | 36.697 | 448.6 | 245.0 | 1.322 | 479.5 | 261.9 | 1.413 | 0.803 | 0.645 | 0.355 | 0.151 | 0.075 | 0.204 | 0.041 | 0.071 | 0.029 | 0.063 | 0.288 |
| N=3 |  | 19.057 ± 2.137 | 0.740 ± 0.070 | 39.141 ± 1.431 | 476.67 ± 32.453 | 268.16 ± 29.376 | 1.411 ± 0.040 | 507.08 ± 28.10 | 284.82 ± 27.51 | 1.506 ± 0.053 | 0.765 ± 0.039 | 0.627 ± 0.031 | 0.373 ± 0.031 | 0.173 ± 0.020 | 0.079 ± 0.005 | 0.199 ± 0.013 | 0.040± 0.000 | 0.076± 0.010 | 0.034 ± 0.005 | 0.049± 0.006 | 0.270± 0.009 |
| 2381 | Adult | 86.00 | 2,820 | 32.791 | 315.1 | 799.1 | 0.929 | 352.6 | 806.5 | 0.938 | 0.811 | 0.717 | 0.283 | 0.151 | 0.068 | 0.133 | 0.037 | 0.051 | 0.017 | 0.028 | 0.211 |
| 2383 | Adult | 65.50 | 2,180 | 33.282 | 347.7 | 681.5 | 1.040 | 428.3 | 784.3 | 1.197 | 0.840 | 0.859 | 0.141 | 0.069 | 0.046 | 0.071 | 0.018 | 0.029 | 0.008 | 0.016 | 0.308 |
| 2350 | Adult | 72.72 | 1.654 | 22.745 | 434.7 | 564.8 | 0.777 | 643.2 | 835.4 | 1.149 | 0.785 | 0.696 | 0.304 | 0.194 | 0.030 | 0.110 | 0.040 | 0.030 | 0.024 | 0.016 | 0.274 |
| N=3 |  | 74.74 ± 4.902 | 2.218 ± 0.275 | 29.606 ± 2.803 | 365.837 ± 29.149 | 681.805 ± 55.241 | 0.915 ± 0.062 | 474.71 ± 71.06 | 808.71 ± 12.07 | 1.095 ± 0.065 | 0.812 ± 0.013 | 0.757 ± 0.042 | 0.243 ± 0.042 | 0.138 ± 0.030 | 0.048 ± 0.009 | 0.105 ± 0.015 | 0.032± 0.005 | 0.037± 0.059 | 0.016 ± 0.004 | 0.020± 0.003 | 0.264± 0.023 |
